# Supplementary material for: Modular self-assembly of gamma-modified peptide nucleic acids in organic solvent mixtures
Source: Nat Commun. 2020 Jun 11;11:2960. doi: 10.1038/s41467-020-16759-8 (PMC7289805; doi:10.1038/s41467-020-16759-8)
Supplement: Supplementary file 1 — Supplementary Information [file 41467_2020_16759_MOESM1_ESM.pdf]

Modular self-assembly of gamma-modified peptide nucleic acids in organic solvent mixtures

Sriram Kumar[1], Alexander Pearse[2], Ying Liu[1], Rebecca E. Taylor[1, 3, 4‡]

[1] Department of Mechanical Engineering, Carnegie Mellon University, Pittsburgh, Pennsylvania

[2] Department of Chemistry, Carnegie Mellon University, Pittsburgh, Pennsylvania

[3] Department of Biomedical Engineering, Carnegie Mellon University, Pittsburgh, Pennsylvania

[4] Department of Electrical and Computer Engineering, Carnegie Mellon University, Pittsburgh, Pennsylvania

‡ Correspondence and requests for materials should be addressed to R.E.T (bex@andrew.cmu.edu)

Supplementary Information

γPNA oligomer name	Sequence	DNA oligomer name	Sequence
P1	N-AATAGCGTTCAC-C	D1	5’-AATAGCGTTCAC-3’
P2	N-GCTATTGAGTAA-C	D2	5’-GCTATTGAGTAA-3’
P3	N-GACATCTTACTC-C	D3	5’-GACATCTTACTC-3’
P4	N-CTGGCGTGCGGA-C	D4	5’-CTGGCGTGCGGA-3’
P5	N-CGCCAGCCCTCG-C	D5	5’-CGCCAGCCCTCG-3’
P6-biotin	N-Biotin-GTGAACCGAGGG-C	D6	5’-GTGAACCGAGGG-3’
P7	N-AGTTTTGATGTC-C	D7	5’-AGTTTTGATGTC-3’
P8-Cy3	N-Cy3-AAAACTACAGAA-C	D8	5’-AAAACTACAGAA-3’
P9	N-TCCGCATTCTGT-C	D9	5’-TCCGCATTCTGT-3’
P2m (mismatch)	N-GCTATTGAGTAAA-C	D3-FAM	5’-GACATCTTACTC-FAM-3’

Supplementary Table 1: Individual γPNA and DNA oligomer sequences used with modifications as indicated. Underlined bases indicate the gamma-position modification on each sequence. Sequences were generated and verified using the DNA Design software developed by the Winfree Lab at Caltech.[1]

aeg-PNA oligomer name	Sequence
aeg-P1	N-AATAGCGTTCAC-C
aeg-P2	N-GCTATTGAGTAA-C
aeg-P3	N-GACATCTTACTC-C
aeg-P4	N-CTGGCGTGCGGA-C
aeg-P5	N-CGCCAGCCCTCG-C
aeg-P7	N-AGTTTTGATGTC-C
aeg-P9	N-TCCGCATTCTGT-C

Supplementary Table 2: Individual aeg-PNA oligomer sequences were synthesized with sequence information as shown in this table.

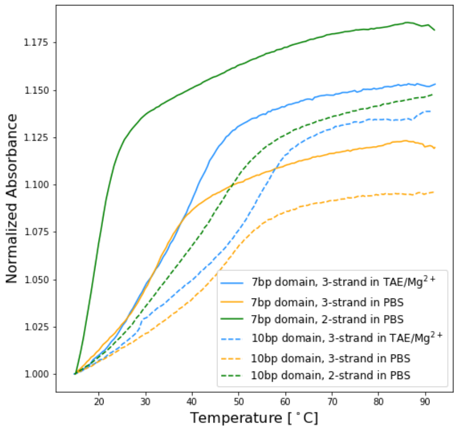

Number of DNA oligomers	Length of domain	Buffer	T_m(°C)
2	7bp	1xPBS	< 20°C
3	7bp	1XPBS	30 ± 1
3	7bp	1X TAE, 12.5mM Mg	37 ± 1
2	10bp	1xPBS	39 ± 1
3	10bp	1XPBS	43 ± 1
3	10bp	1X TAE, 12.5mM Mg	28 ± 1<br>50 ± 1

Supplementary Figure 1: DNA UV melting curves in physiological and origami buffers. UV-melting studies in PBS (orange and red curves) and 1X TAE,12.5mM Mg^{2+} (blue curves) of 2-oligomer and 3-oligomer DNA systems. Data sets demonstrate that the maximum melting temperatures (T_m) for 7-base overlaps (solid curves) is 37 °C (3-oligomer, TAE-Mg buffer) and for 10-base overlaps (dashed curves) is 50 °C (3-oligomer, TAE-Mg buffer).10-base pair domain and 7-base pair domain sequences were chosen from Hariadi et al.[2] and Yang et al.[3] Source data are provided as a Source Data file.

Helix	No. of oligomers	Oligomer strand ID	Buffer/solvent	T_m (°C)
A	2	P2, P3	1X PBS	39 ± 1
A	3	P2, P3, P7	1X PBS	28 ± 1,<br>59 ± 1
A	3	P2, P3, P7	DMF	51 ± 1
A	3	P2, P3, P7	DMSO	54 ± 1
B	2	P4, P5	1X PBS	43 ± 1
B	3	P4, P5, P6	1X PBS	53 ± 1
B	3	P4, P5, P6	DMF	50 ± 1
C	2	P8, P9	1X PBS	56 ± 1
C	3	P7, P8, P9	1X PBS	63 ± 1
C	3	P7, P8, P9	DMF	56 ± 1

Supplementary Table 3: Thermodynamic parameters from UV melting curve analysis of 2- and 3- oligomer γPNA systems indicating both trends in cooperativity and reasonable thermal stability of γPNA duplexes in organic solvents. Source data are provided as a Source Data file.

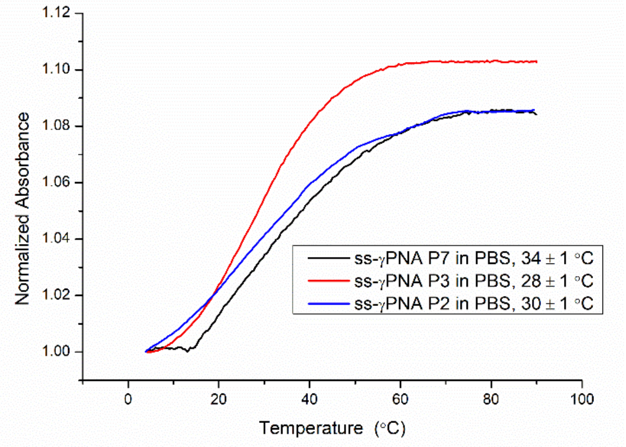

Supplementary Figure 2: UV melting curves of single-stranded γPNA oligomers (ss- γPNA) in aqueous buffer. Melt curves of ss- γPNA oligomers P2 (blue curve), P3 (red curve) and P7 (black curve) with T_m between 28 and 34 °C. Source data are provided as a Source Data file.

Temperature Range	Temperature change	Time interval per temperature change
90-80 °C	0.1°C	1 min
80-70 °C	0.1°C	1 min
70-60 °C	0.1°C	3 min
60-50 °C	0.1°C	3 min
50-40 °C	0.1°C	3 min
40-30 °C	0.1°C	1 min
30-20 °C	0.1°C	1 min
4 °C	0.1°C	Hold

Supplementary Table 4: Temperature ramp protocol for γPNA nanotubes.

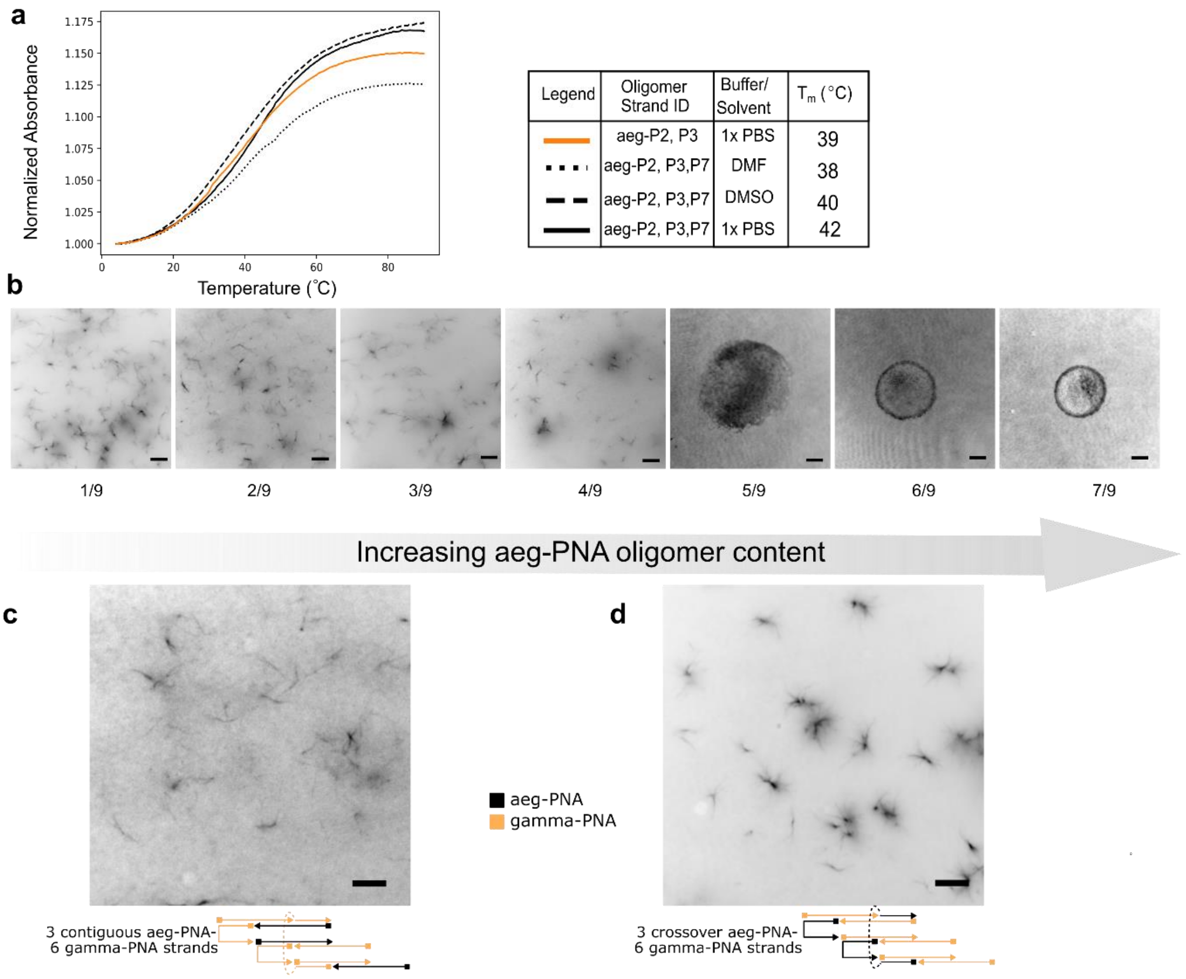

Supplementary Figure 3: Melt curve and TIRF assays of aeg-PNA oligomers. a) Melt curve studies show the melting temperatures of 2-oligomer (orange curves) and 3-oligomer (black curves) substructures in different solvent conditions varies between 39 to 42 °C. This indicates that 6-base domains are sufficiently stable for aeg-PNA duplex systems in PBS (solid curve), DMSO (dashed curve) and DMF (dotted curves). Source data are provided as a Source Data file. **b)** TIRF panels (5 µm scale bar) of the self-assemblies formed by aeg-PNA-γPNA hybrids in 75% DMSO: H_2O ($V V^{-1}$) with increasing content of aeg-PNA oligomer content as indicated (repeated over 2 independent experiments for multiple combinations for each condition). Formation of aggregates are visible under TIRF microscopy when the overall aeg-PNA content is more than 50%. **c-d)** TIRF characterization (5 µm scale bar) of γPNA-aeg-PNA hybrid filaments through selective replacement of γPNA oligomers with aeg-PNA (repeated over 2 independent experiments for each condition). Schematic representations show the position in the SST motif replaced with aeg-PNA (black arrows) in the context of other γPNA sequences (orange arrows). Sequential replacement of **c)** 3 contiguous γPNA sequence with aeg-PNA show no discernible morphological change and **d)** 3 crossover γPNA sequences with aeg-PNA resulted in stellate structures with pronounced bundling effects.

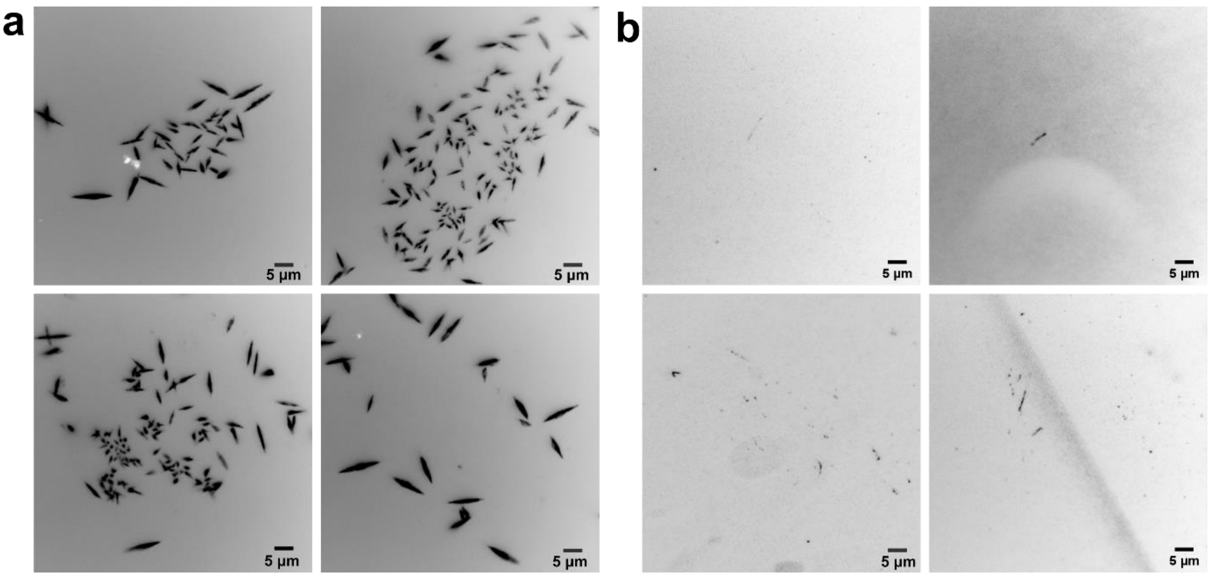

Supplementary Figure 4: TIRF assay characterization of different morphologies for self-assembling γPNA oligomers in DMF and 1,4-dioxane solvent mixtures a) TIRF microscopy images (5 µm scale bar) of self-assembly of γPNA oligomers in 75% DMF: H_2O ($V\ V^{-1}$) showing needle-like nanostructures (repeated over 3 independent experiments) **b)** TIRF microscopy images (5 µm scale bar) of self-assembly of γPNA oligomers in 40% 1,4 dioxane: H_2O ($V\ V^{-1}$) showing sparse decoration of filamentous nanostructures (repeated over 2 independent experiments)

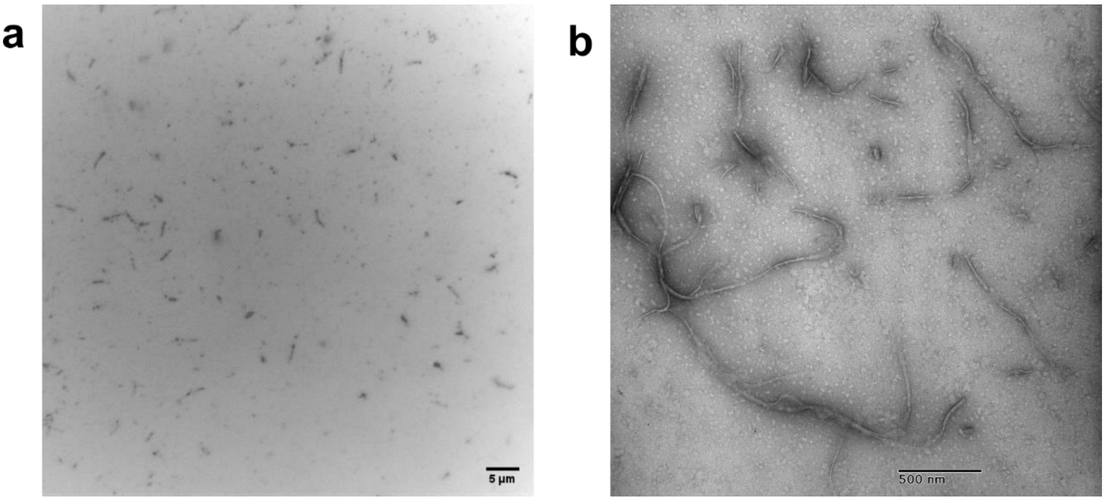

Supplementary Figure 5: TIRF and TEM data of self-assembled γPNA nanotubes in 75% DMSO: H_2O a) Wide view field image (5 µm scale bar) of γPNA nanotubes visualized using a TIRF microscopy while monitoring the 561 nm laser line (Cy3) (repeated over 3 independent experiments). **b)** TEM images (scale bar 500 nm) of γPNA nanotubes visualized under low magnification (repeated over 4 independent experiments).

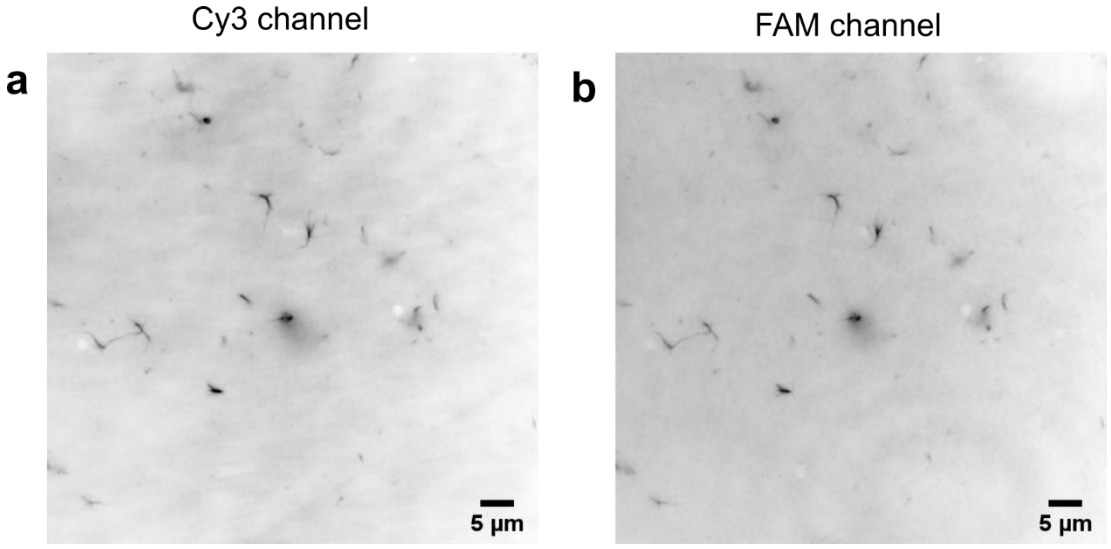

Supplementary Figure 6: TIRF co-localization study of self-assembled γPNA-DNA nanotubes in 75% DMSO: H_2O. Evidence of nanotube formation on replacing one γPNA oligomer (P3) with a FAM-labeled DNA oligomer (D3-FAM) through dye-colocalization studies (repeated over 3 independent experiments). Image (5 µm scale bar) shows colocalized dyes on tubular constructs when viewed under Cy3 channel (**a**) and the FAM channel (**b**) indicating that constructs continue to self-assemble upon introduction of a dye-labelled DNA oligomer.

Supplementary note 1: Contour length measurements of γPNA 3-helix tube using TIRF microscopy.

Contour length measurements of the nanotubes was carried out using TIRF microscopy to achieve a larger field of view enabling more data sampling and collection. The length resolution in our measurements is limited by pixel resolution at 120 nm/pixel for a 60x objective with 1.5x magnification. Additionally, we applied a threshold of 2 µm as a length threshold for micron-scale filamentous structures rather than micro-aggregates of SST motifs.

Contour length profile measurements were made from TIRF microscopy images of γPNA and γPNA-DNA hybrids that were decorated with Cy3 fluorophores. Length measurements were made using the NeuronJ plugin (Meijering et al.)[4] for ImageJ.[5]

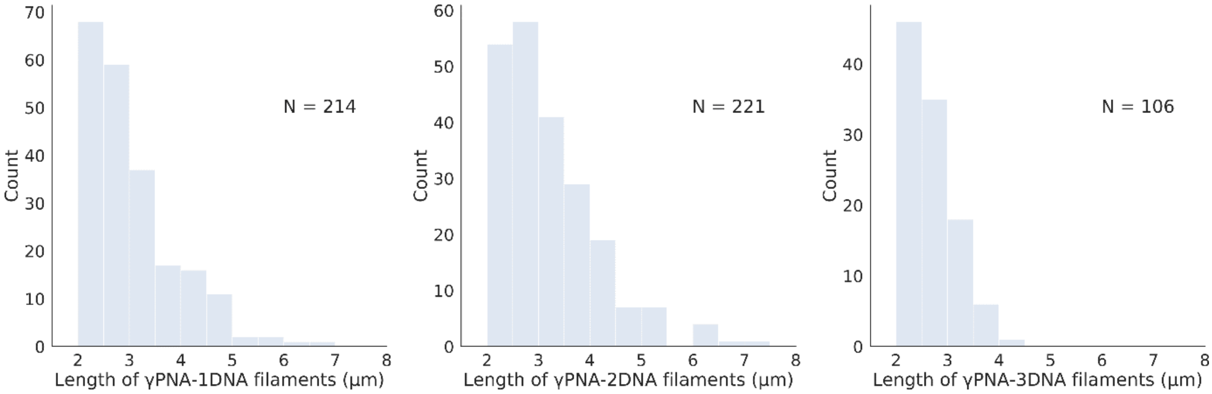

Supplementary Figure 7: Length profiles of self-assembled γPNA-DNA hybrid nanotubes (Left to Right) Length profile histograms of 3-helix γPNA-DNA hybrid tubes self-assembled in 75% DMSO: H_2O introducing 1 contiguous DNA oligomer (left, D3 oligomer, N= 214 over 2 independent experiments), 2 contiguous DNA oligomers (center, oligomers - D3 and D5, N= 221 over 2 independent experiments) and 3 contiguous DNA oligomers (right, oligomers- D3, D5 and D9, N= 106 over 2 independent experiments) obtained from TIRF assay data sets. Analyses shows that tendencies for γPNA-DNA hybrid systems to grow beyond 2 μm reduces because of lower stabilities associated with γPNA-DNA duplexes in organic solvent mixtures. Source data are provided as a Source Data file.

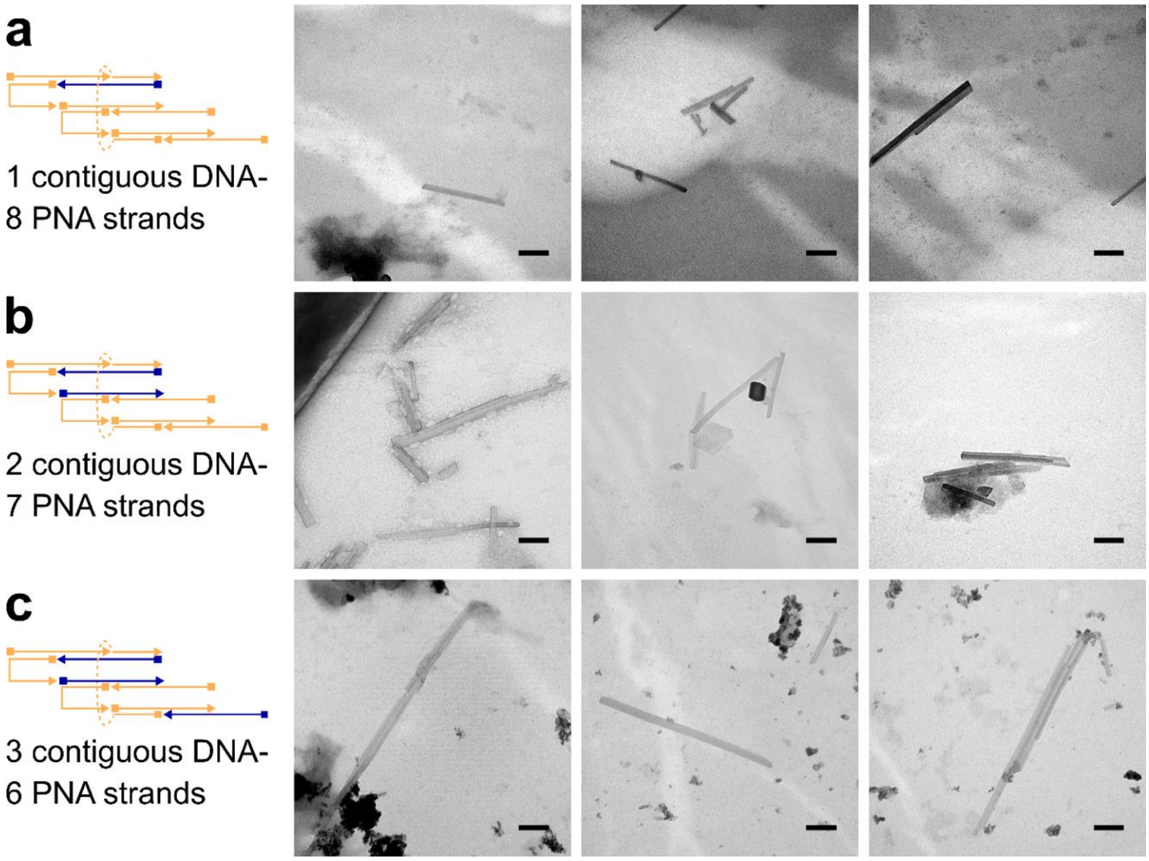

Supplementary Figure 8: TEM images of replacing contiguous γPNA oligomers with DNA. TEM image panel (scale bar 100 nm) shows that sequential replacement of contiguous γPNA by introducing **a**) 1 contiguous DNA oligomer, **b**) 2 contiguous DNA oligomers and **c**) 3 contiguous DNA oligomers resulted in straight filaments (repeated over 2 independent experiments for each condition).

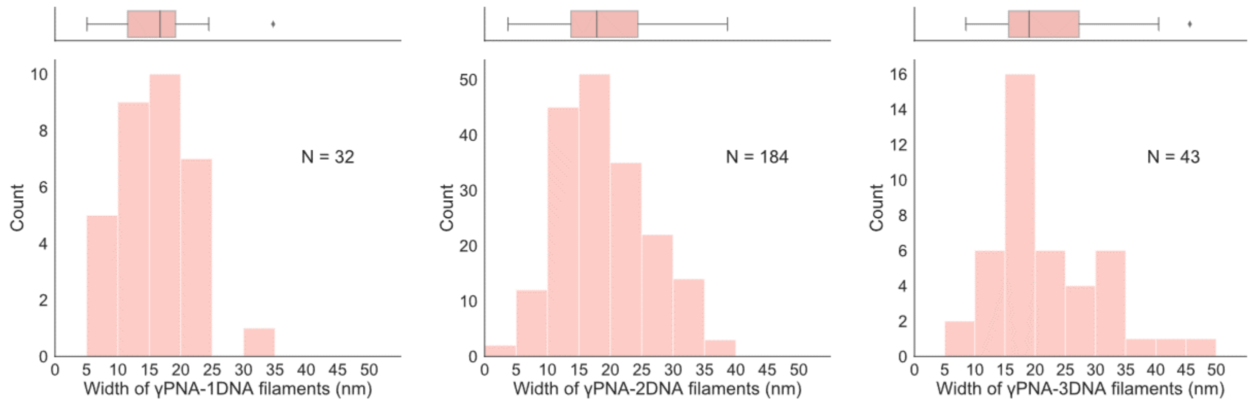

Supplementary Figure 9: Width profiles of self-assembled γPNA-DNA hybrid nanotubes from TEM images (Left to Right). Width histograms of 3-helix γPNA-DNA hybrid tubes self-assembled in 75% DMSO: H_2O introducing 1 contiguous DNA oligomer (left, D3 oligomer, N= 32), 2 contiguous DNA oligomers (center, oligomers - D3 and D5, N= 184) and 3 contiguous DNA oligomers (right, oligomers- D3, D5 and D9, N= 43) obtained from TEM experiments. Median widths were estimated at 15.5 nm (IQR – 8.9 nm), 17.9 nm (IQR – 10.8 nm) and 19 nm (IQR – 12.2 nm) for 1, 2 and 3 contiguous DNA oligomer substitutions, respectively. No statistically significant difference between all 4 constructs could be reported. Source data are provided as a Source Data file. (repeated over 2 independent experiments for each condition)

Supplementary note 2: Width measurements and line profile analysis across the width of 'bundled' γPNA 3-helix tubes using TEM data. TEM images of the designed γPNA nanotubes bundle revealed multiple dark striated regions (a cavity or hole) flanked by brighter regions (γPNA) along the middle of our constructs. This contrast occurs as uranyl acetate staining can fill only the space around the γPNA constructs. An intensity profile along the filament's cross section confirmed the existence of multiple cavities which appear as alternating bands of light and dark under TEM, suggesting bundling on nanotubes.

Additionally, the intensity profile scans provide full widths across the bundled nanotubes cross-section. The scan and analysis were performed via ImageJ.[5]

Median widths for individual constructs were determined using quartile ranges. Outliers were excluded using interquartile range. Mann-Whitney tests were performed between all-γPNA construct and each γPNA-DNA construct to compare width measurements to verify any statistically significant difference.

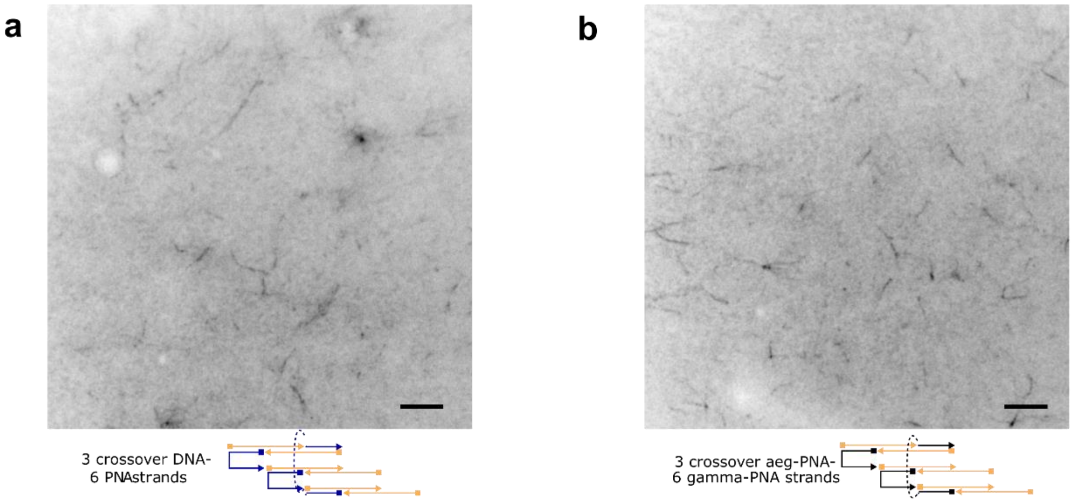

Supplementary Figure 10: TIRF assay images on the effect of SDS towards reduced bundling in crossover oligomer replacements. a) TIRF (5 µm scale bar) images of 3 DNA oligomer crossover replacements in the presence of 5.25 mM SDS during self-assembly shows disappearance of stellate-like morphology (repeated over 2 independent experiments). **b)** Similar effects are seen when TIRF (5 µm scale bar) assays are performed with the replacement of 3 aeg-PNA oligomer crossover replacements in the presence of 5.25 mM SDS during self-assembly (repeated over 2 independent experiments). This is indicative of the capability of anionic surfactants like SDS to counter increased non-specific interactions between hybrid nanotube structures promoted by hydrophobic effects.

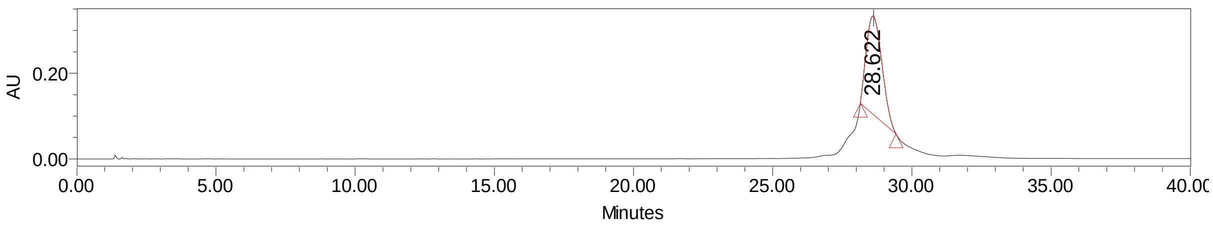
AU
0.20
0.00
28.622
0.00
5.00
10.00
15.00
20.00
25.00
30.00
35.00
40.00
Minutes

(a)

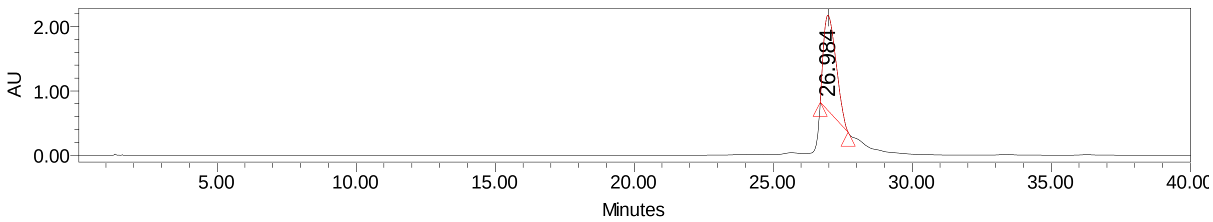
AU
2.00
1.00
0.00
26.984
5.00
10.00
15.00
20.00
25.00
30.00
35.00
40.00
Minutes

(b)

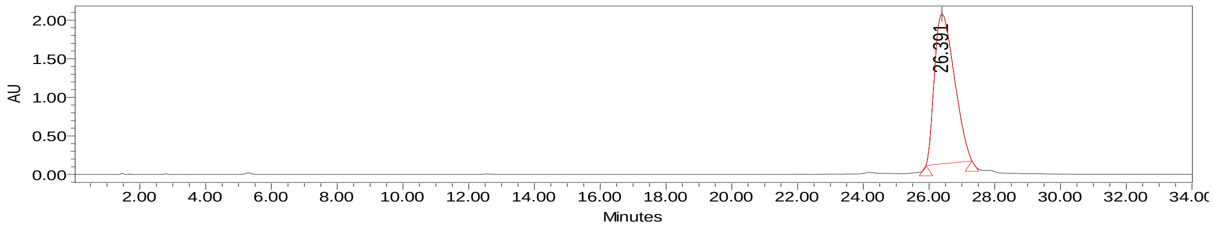
AU
2.00
1.50
1.00
0.50
0.00
26.391
2.00
4.00
6.00
8.00
10.00
12.00
14.00
16.00
18.00
20.00
22.00
24.00
26.00
28.00
30.00
32.00
34.00
Minutes

(c)

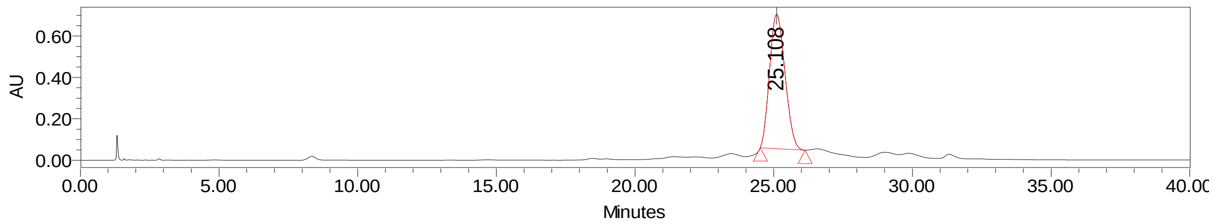
AU
0.60
0.40
0.20
0.00
25.108
0.00
5.00
10.00
15.00
20.00
25.00
30.00
35.00
40.00
Minutes

(d)

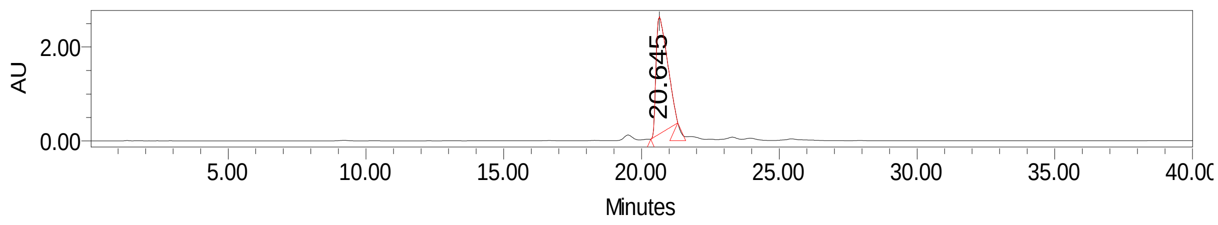

(e)

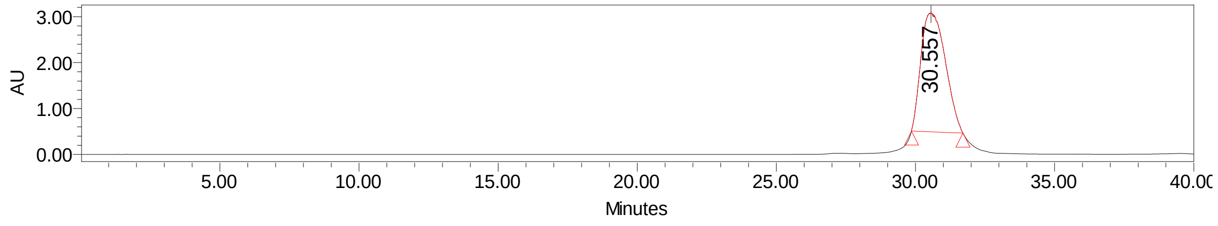

(f)

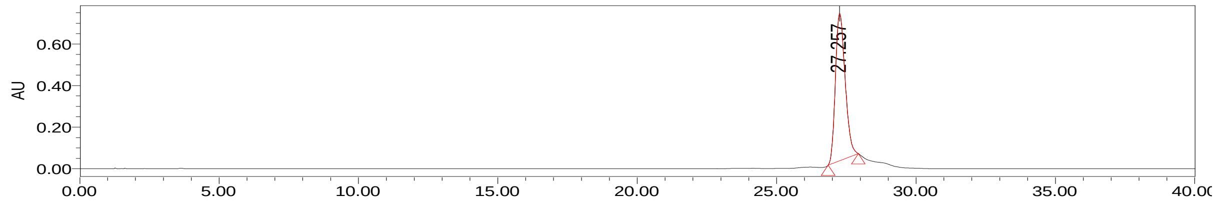

(g)

Supplementary Figure 11: HPLC Chromatogram for (a) aegP1, (b) aegP2, (c) aegP3, (d) aegP4, (e) aegP5, (f) aegP7, (g) aegP9.

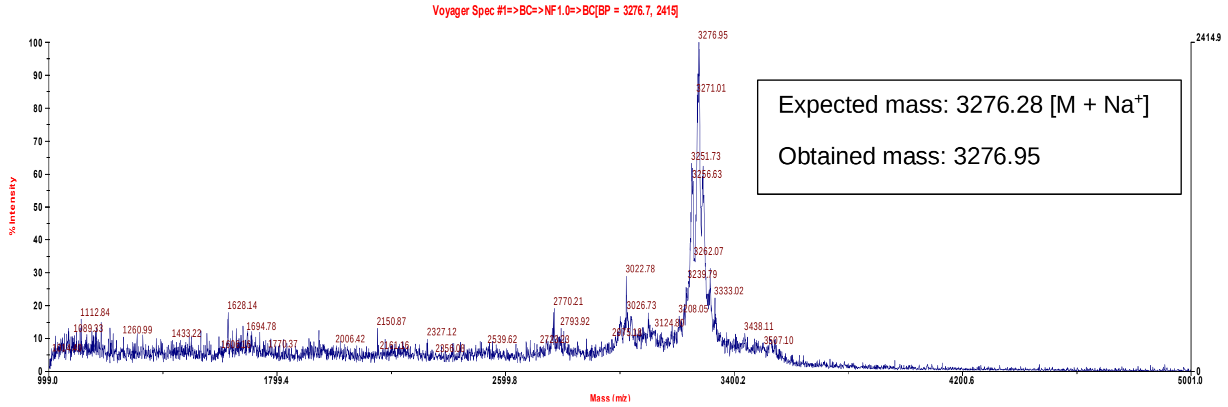
Voyager Spec #1=>BC=>NF1.0=>BC[BP = 3276.7, 2415]
Expected mass: 3276.28 [M + Na+]
Obtained mass: 3276.95
% Intensity
Mass (m/z)

(a)

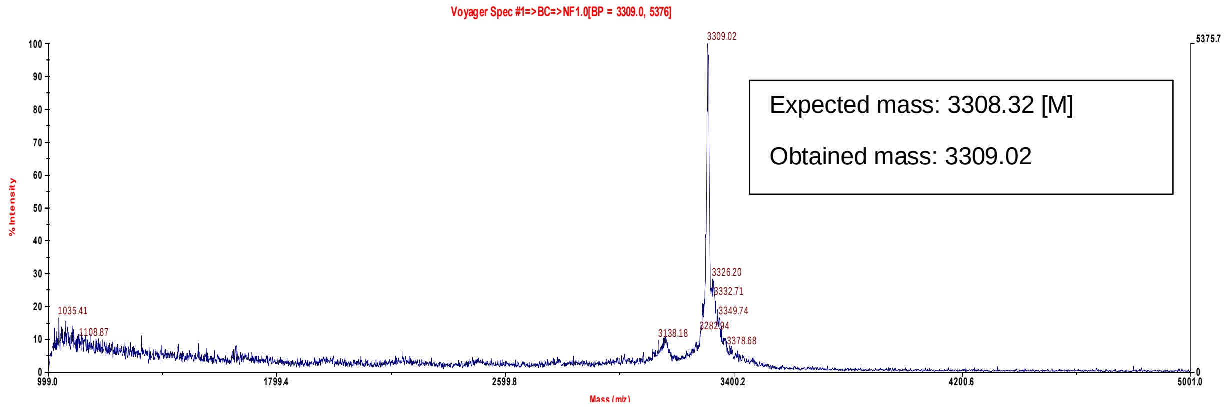
Voyager Spec #1=>BC=>NF1.0[BP = 3309.0, 5376]
Expected mass: 3308.32 [M]
Obtained mass: 3309.02
% Intensity
Mass (m/z)

(b)

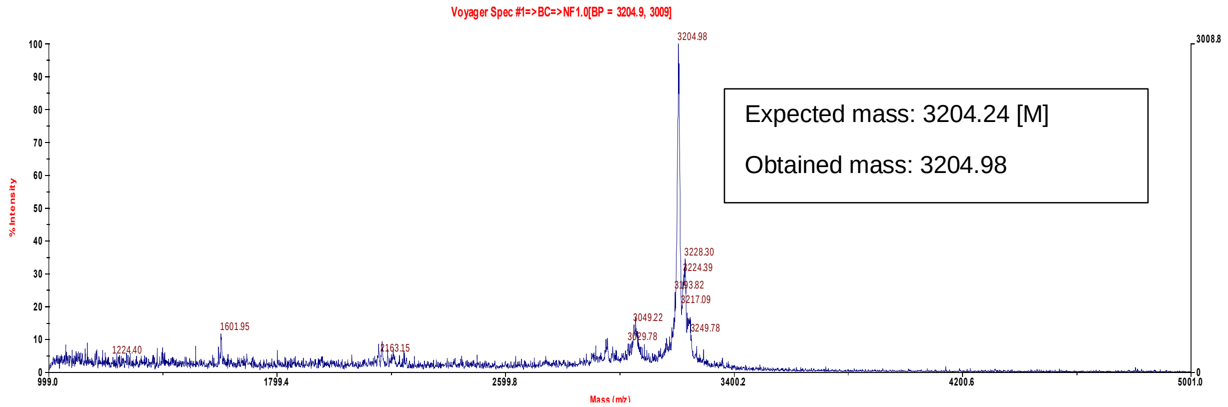
Voyager Spec #1=>BC=>NF1.0[BP = 3204.9, 3009]
Expected mass: 3204.24 [M]
Obtained mass: 3204.98
% Intensity
Mass (m/z)

(c)

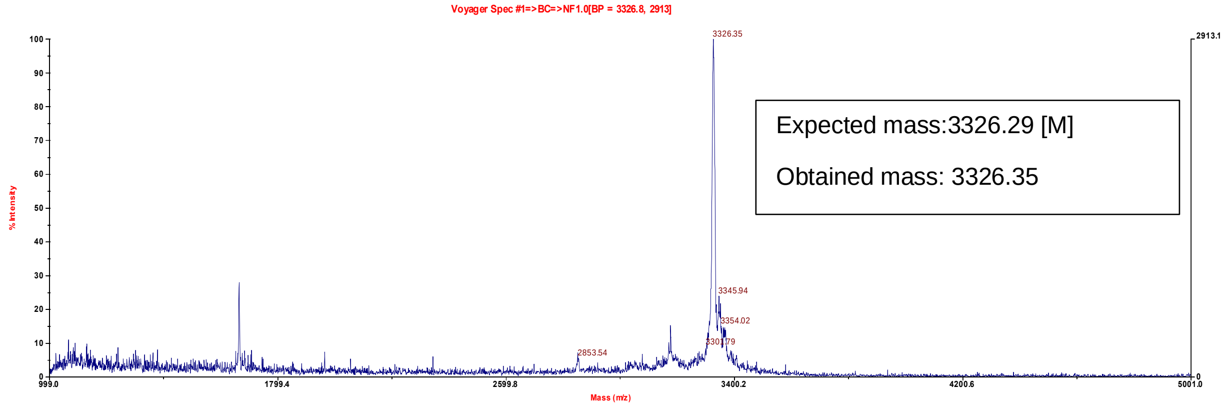
Voyager Spec #1=>BC=>NF1.0[BP = 3326.8, 2913]
Expected mass:3326.29 [M]
Obtained mass: 3326.35
% Intensity
Mass (m/z)

(d)

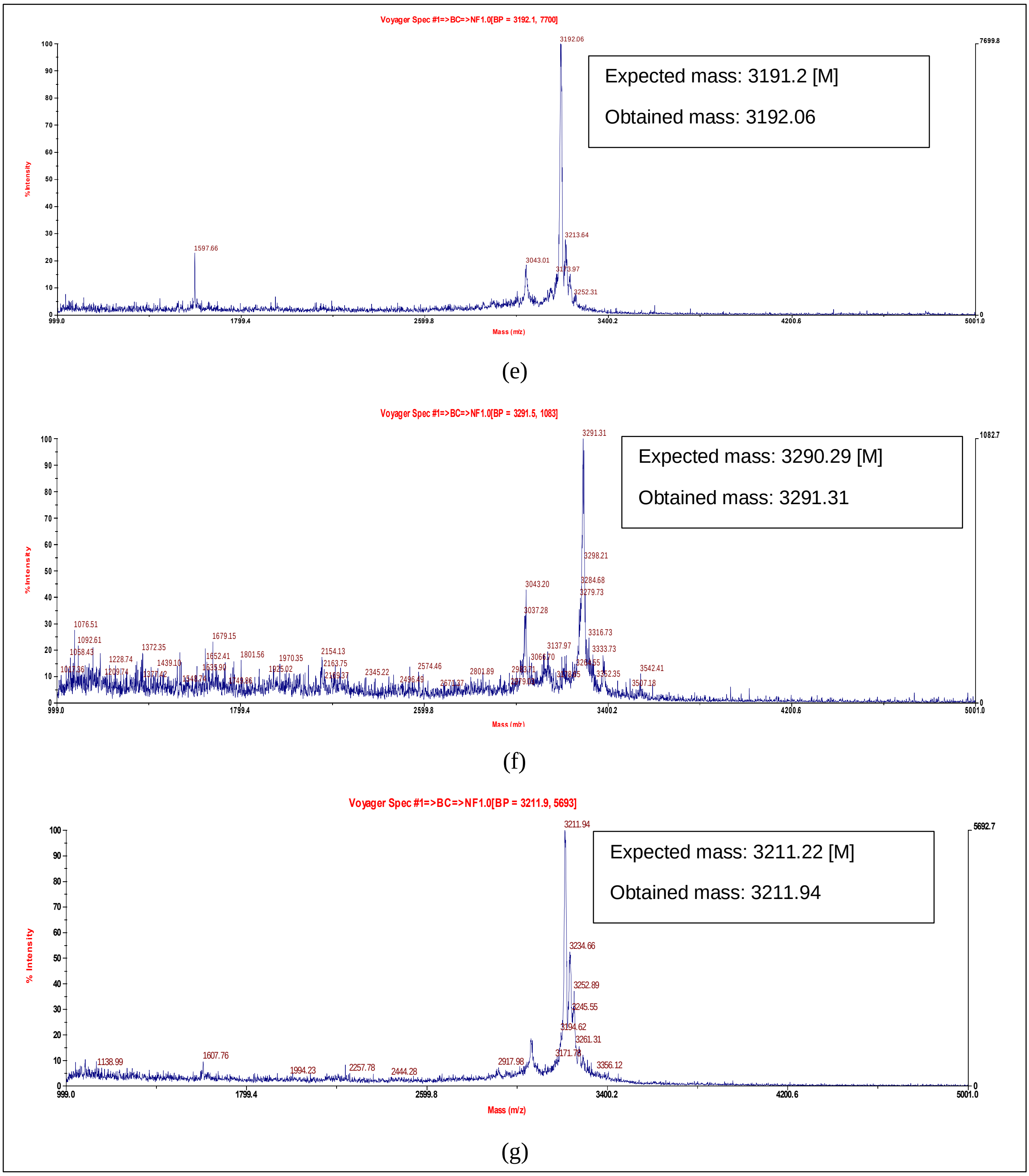

Supplementary Figure 12: MALDI-ToF spectra for (a) aeg-P1, (b) aeg-P2, (c) aeg-P3, (d) aeg-P4, (e) aeg-P5, (f) aeg-P7, (g) aeg-P9.

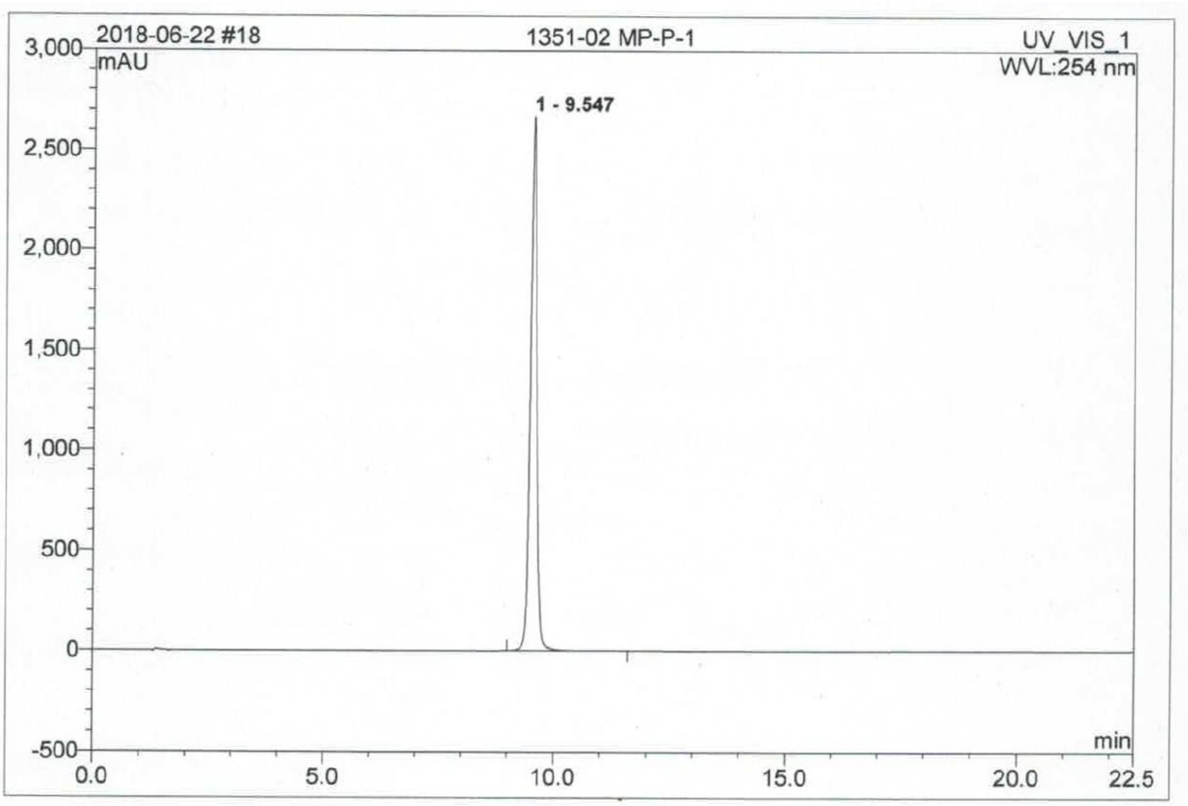

Supplementary Figure 13. HPLC chromatogram for mini-PEG γ-modified oligomer P1.

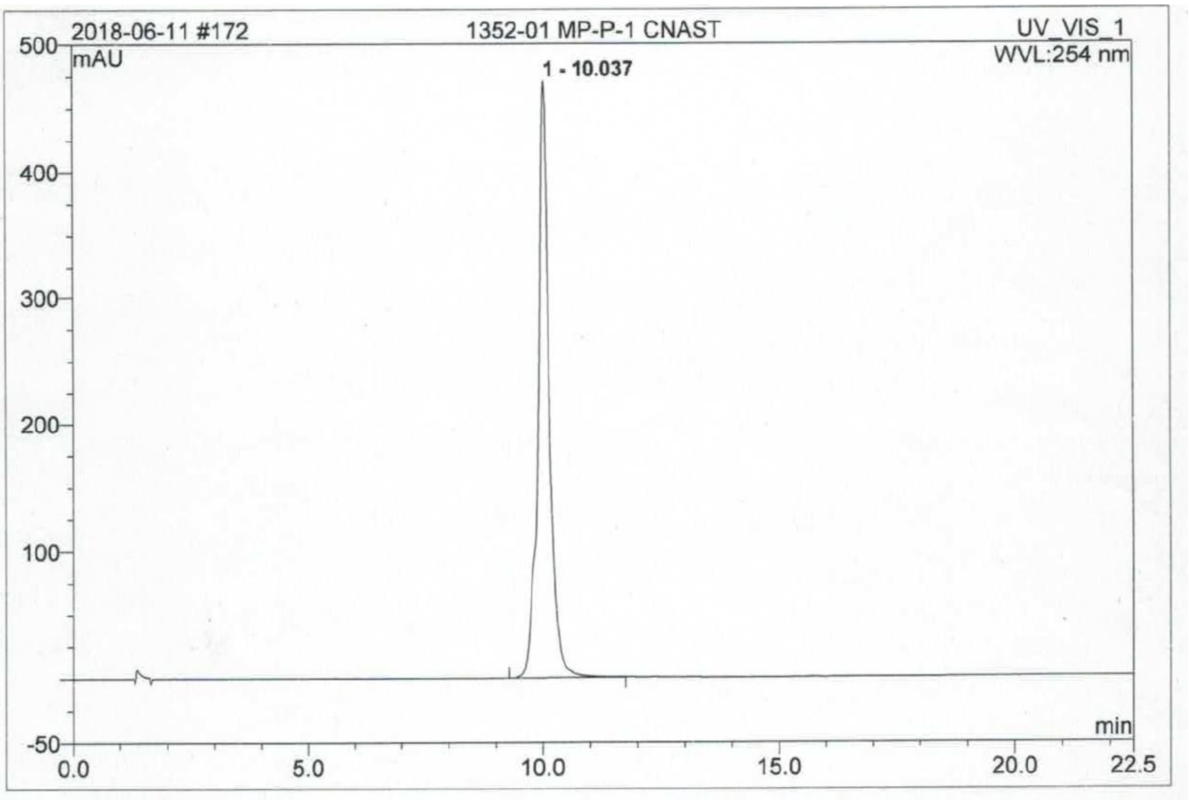

Supplementary Figure 14. HPLC chromatogram for mini-PEG γ-modified oligomer P2.

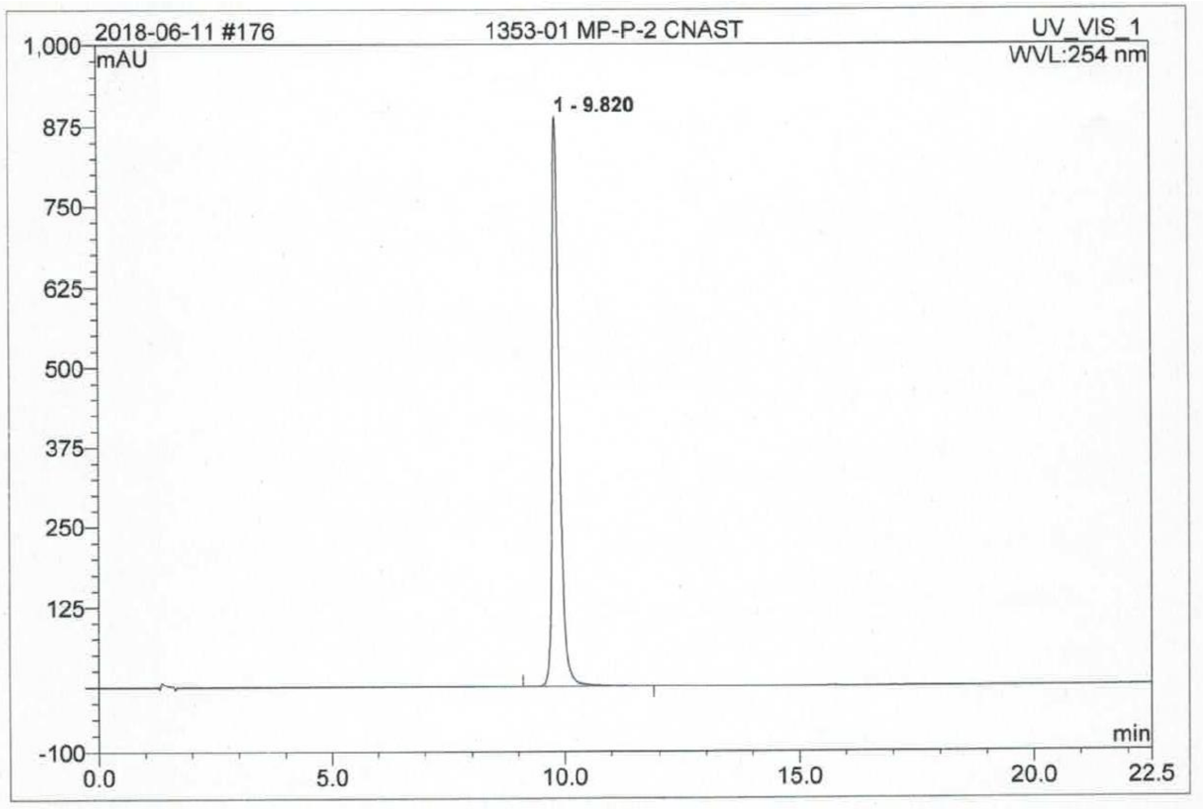

Supplementary Figure 15. HPLC chromatogram for mini-PEG γ-modified oligomer P3.

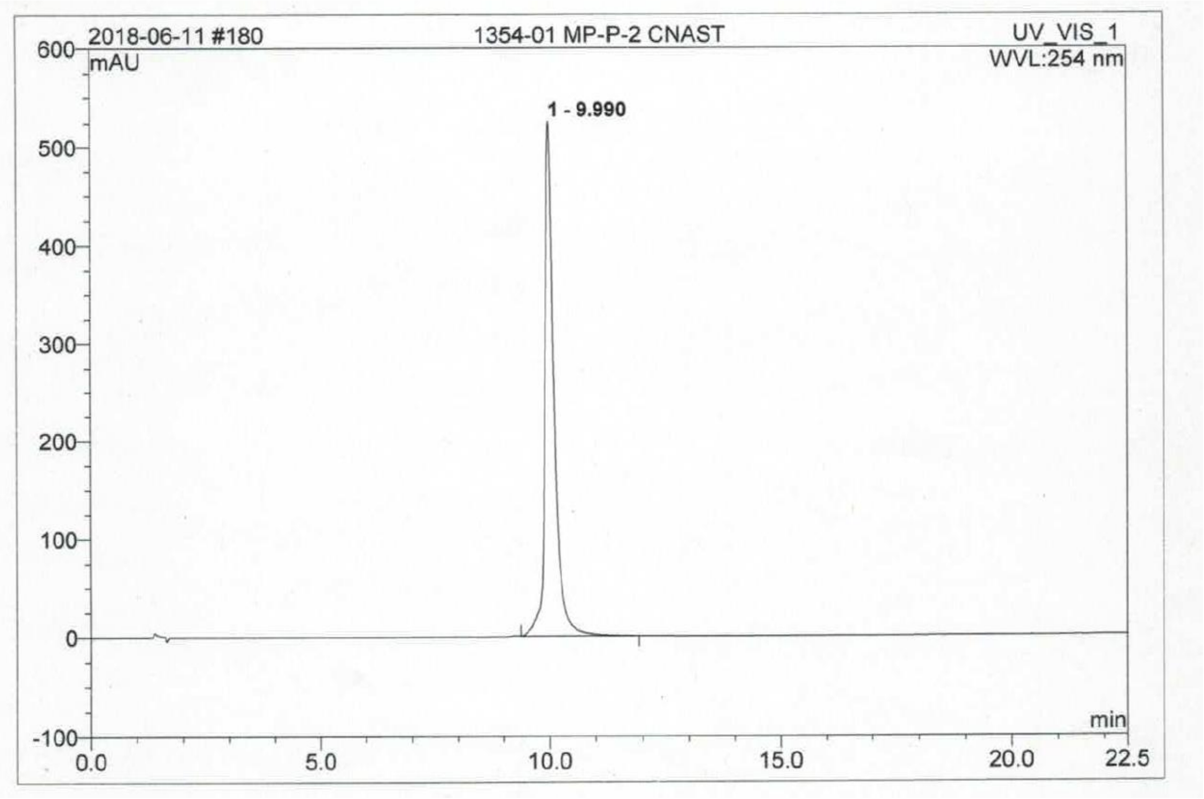

Supplementary Figure 16. HPLC chromatogram for mini-PEG γ-modified oligomer P4.

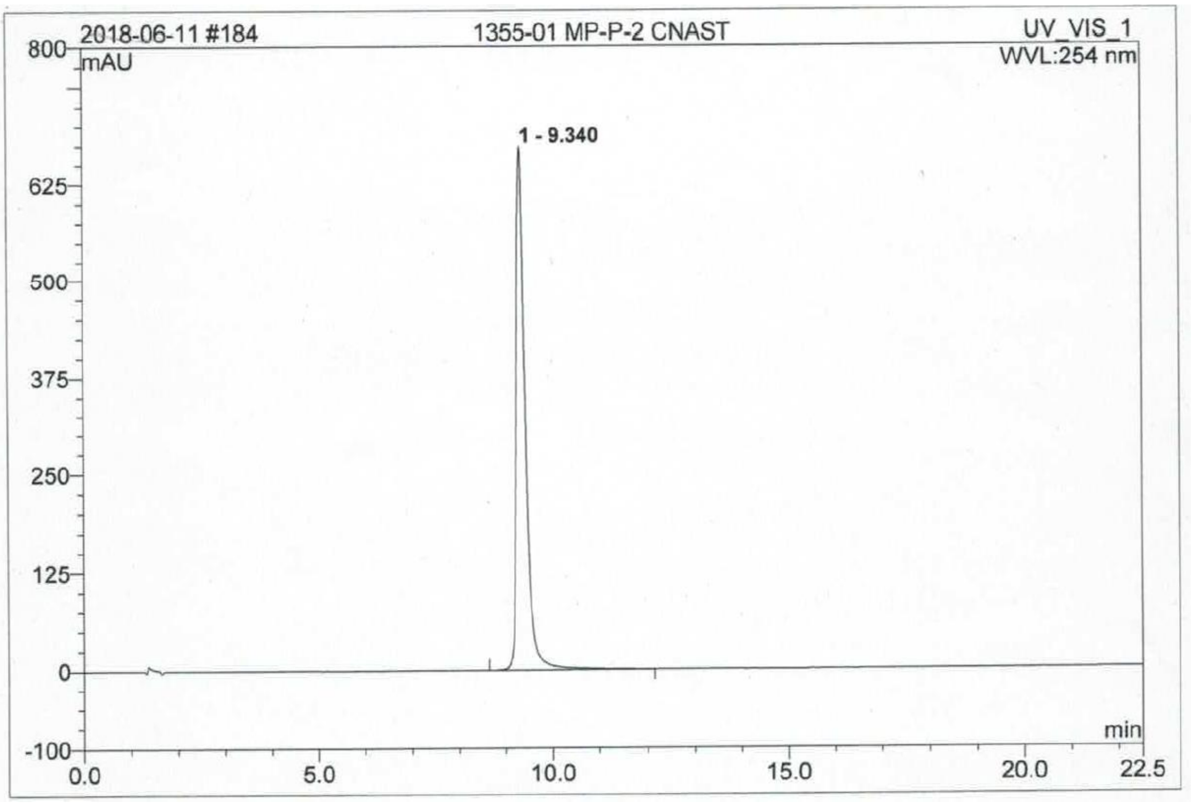

Supplementary Figure 17. HPLC chromatogram for mini-PEG γ-modified oligomer P5.

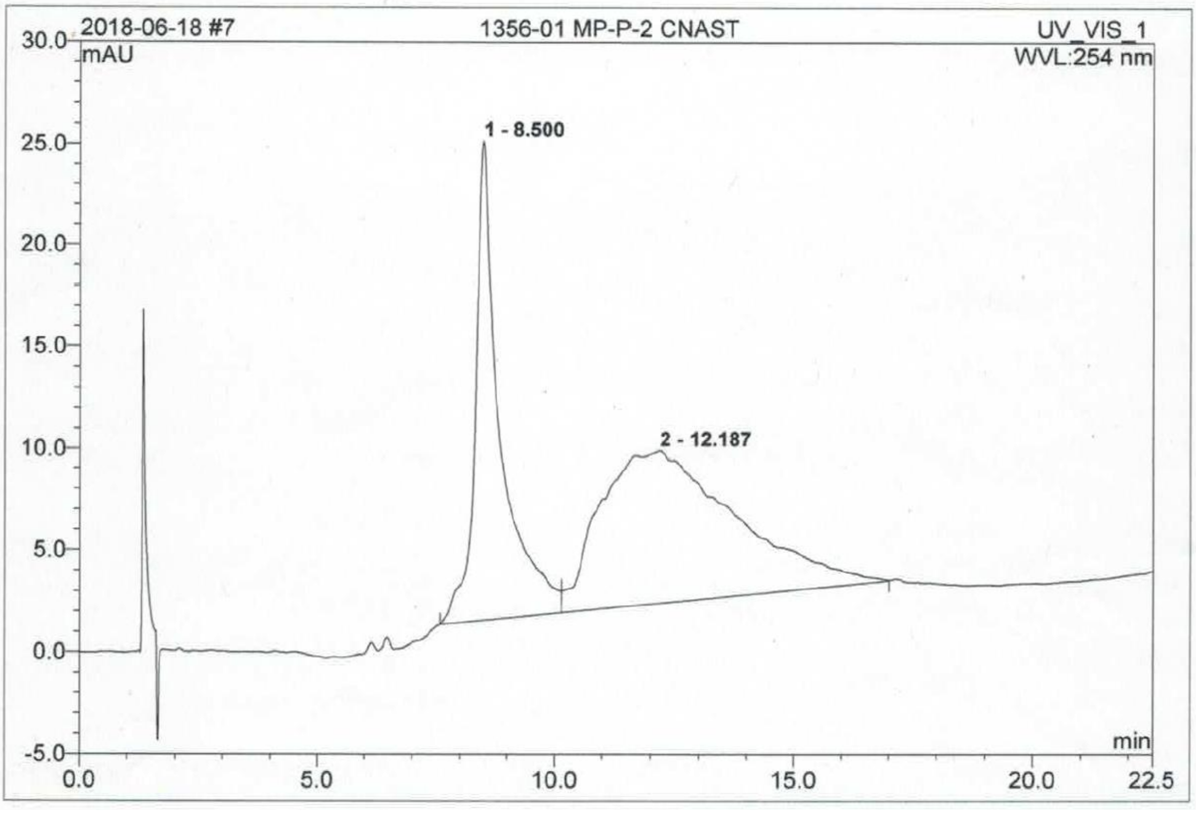

Supplementary Figure 18. HPLC chromatogram for mini-PEG γ-modified oligomer P6-biotin. Chromatogram shows desired peak with an extended shoulder indicating

possible aggregation of P6-biotin. ESI-MS of the corresponding oligomer, however, shows a clean spectrum with the desired mass (Supplementary Figure 29).

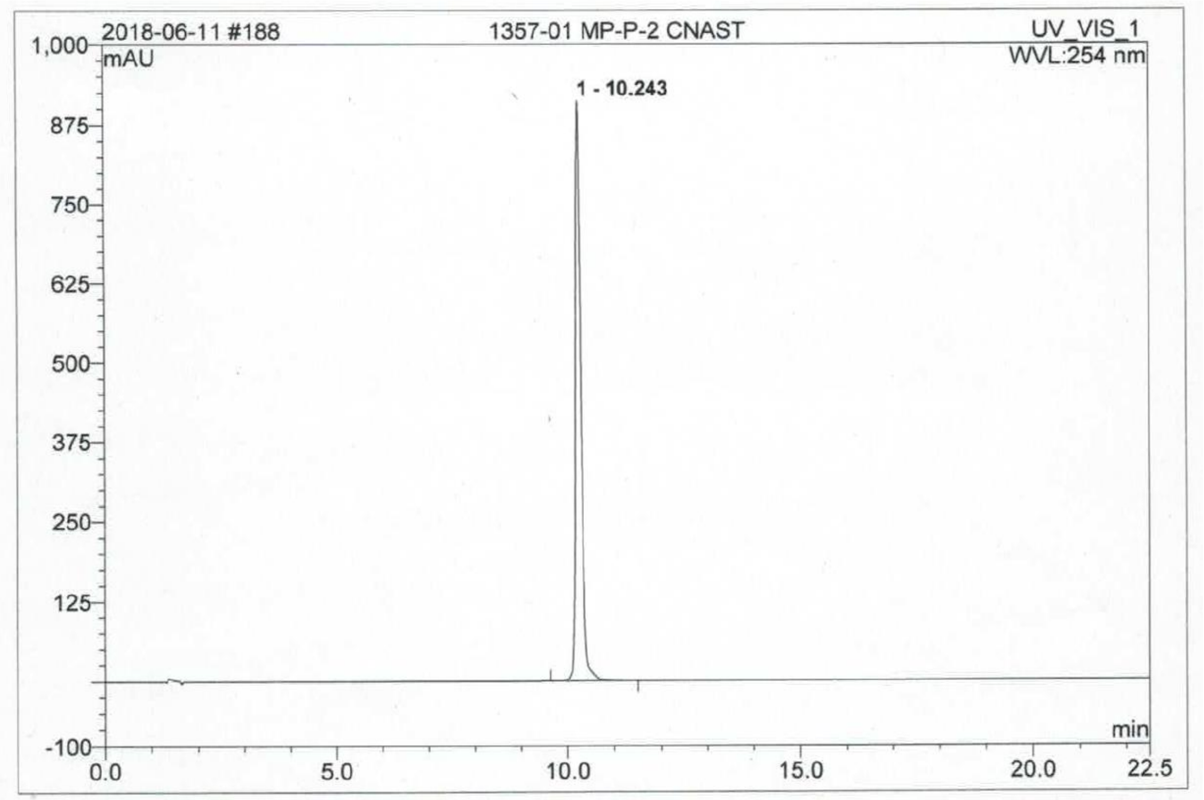

Supplementary Figure 19. HPLC chromatogram for mini-PEG γ-modified oligomer P7.

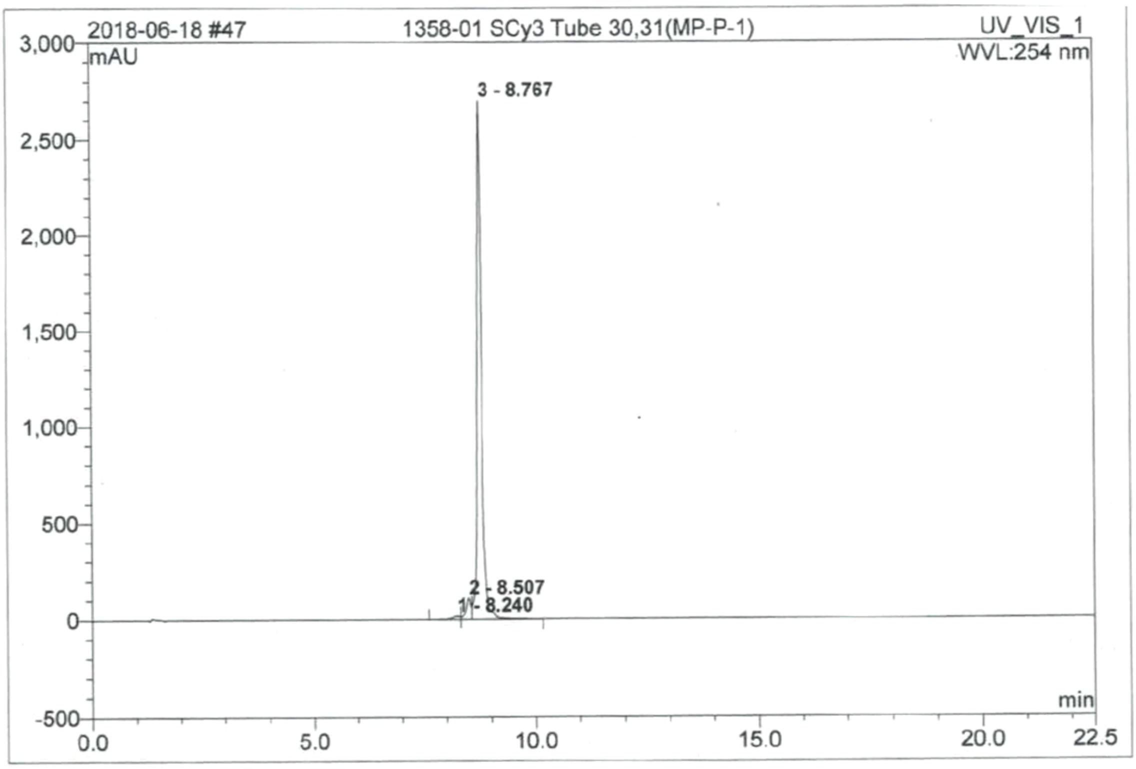

Supplementary Figure 20. HPLC chromatogram for mini-PEG γ-modified oligomer P8-Cy3 (monitored at 254 nm).

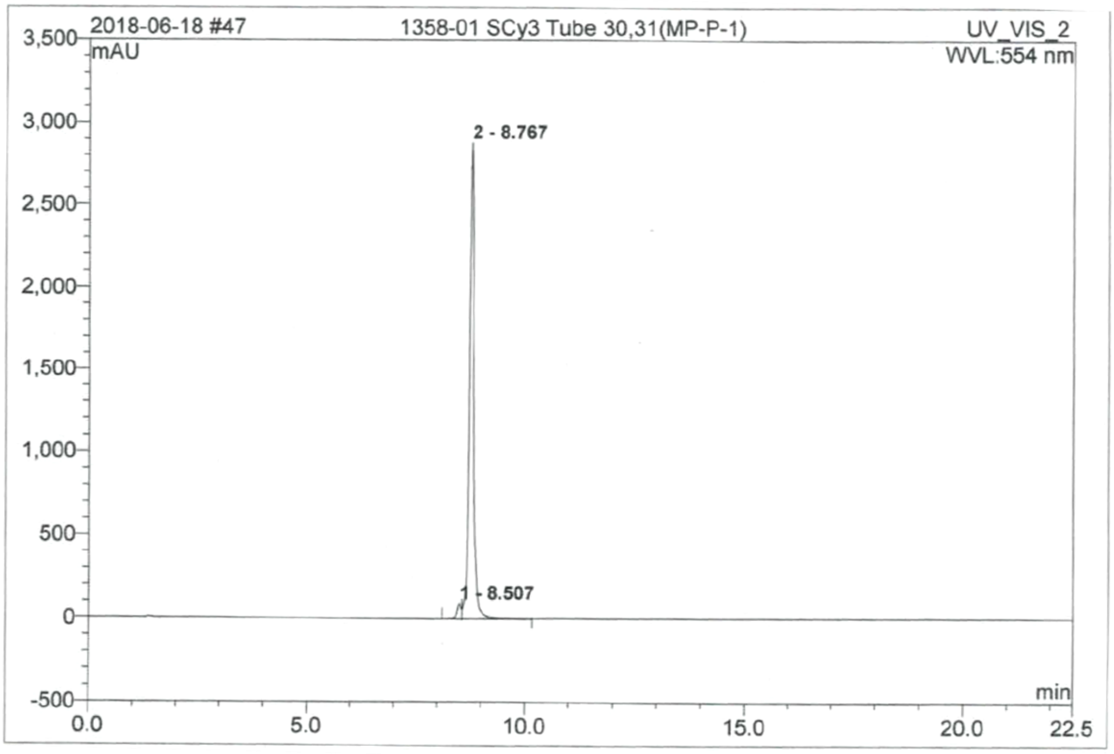

Supplementary Figure 21. HPLC chromatogram for mini-PEG γ-modified oligomer P8-Cy3 (monitored at 554 nm).

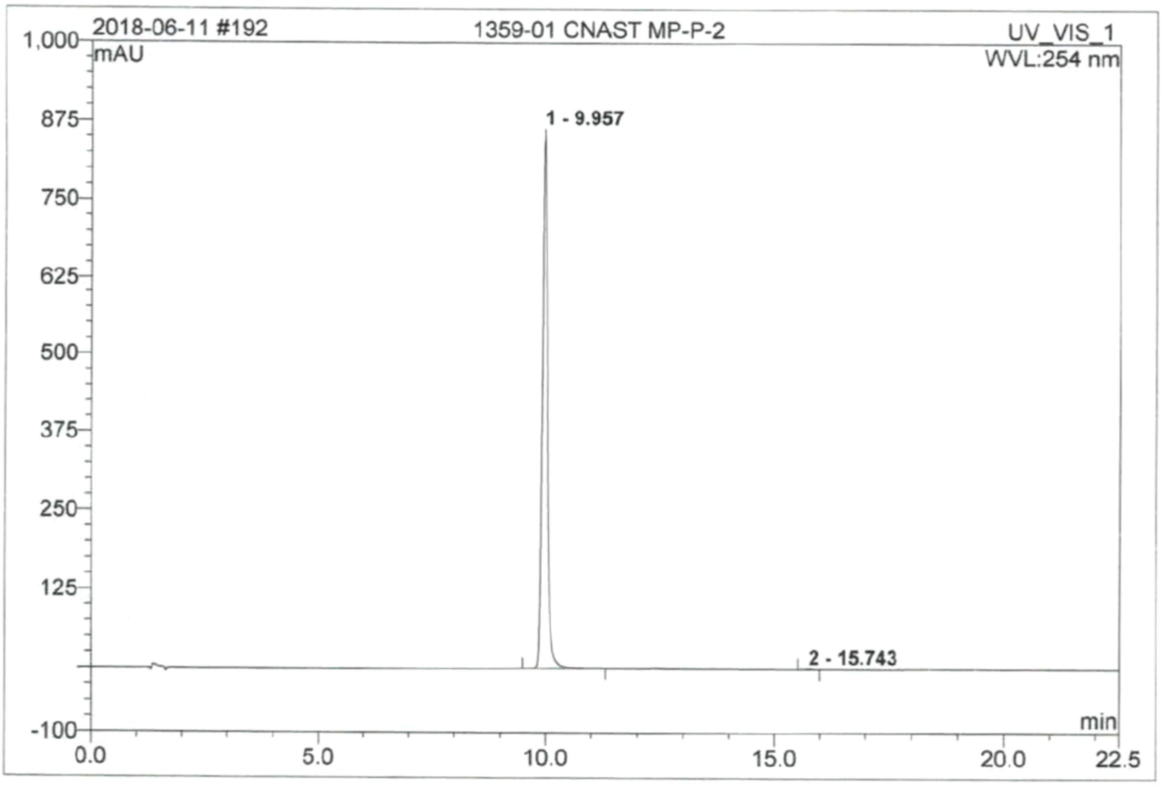

Supplementary Figure 22. HPLC chromatogram for mini-PEG γ-modified oligomer P9.

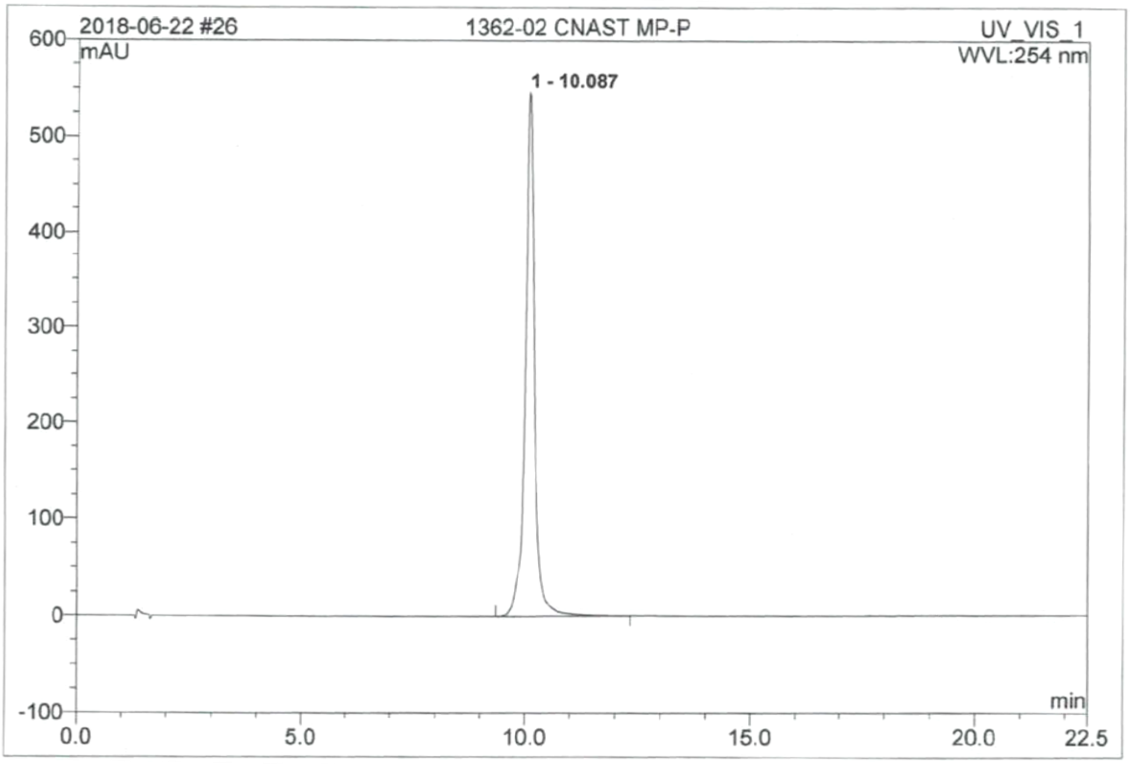

Supplementary Figure 23. HPLC chromatogram for mini-PEG γ-modified oligomer P2m.

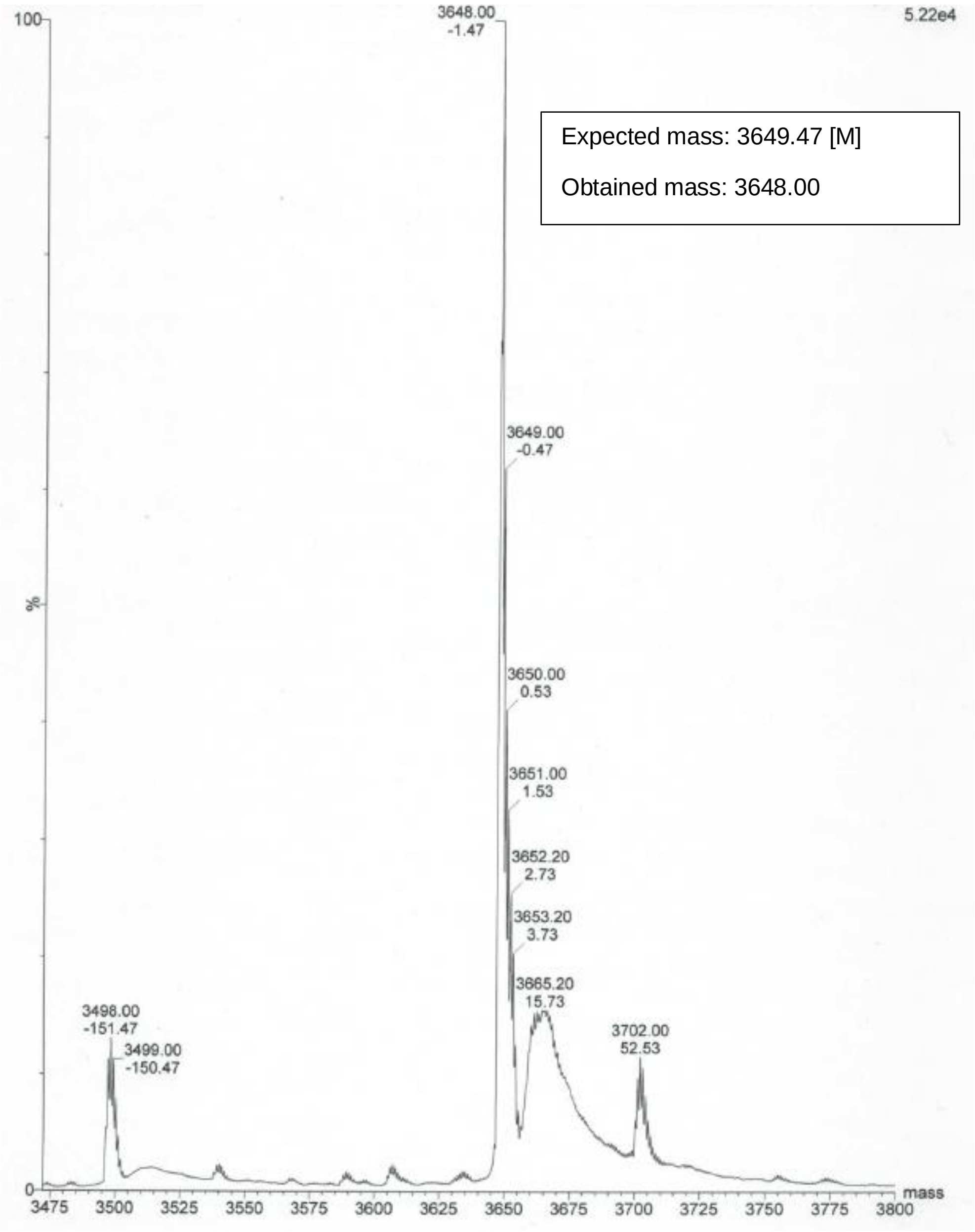

Supplementary Figure 24. ESI-ToF mass spectra for mini-PEG γ-modified oligomer P1.

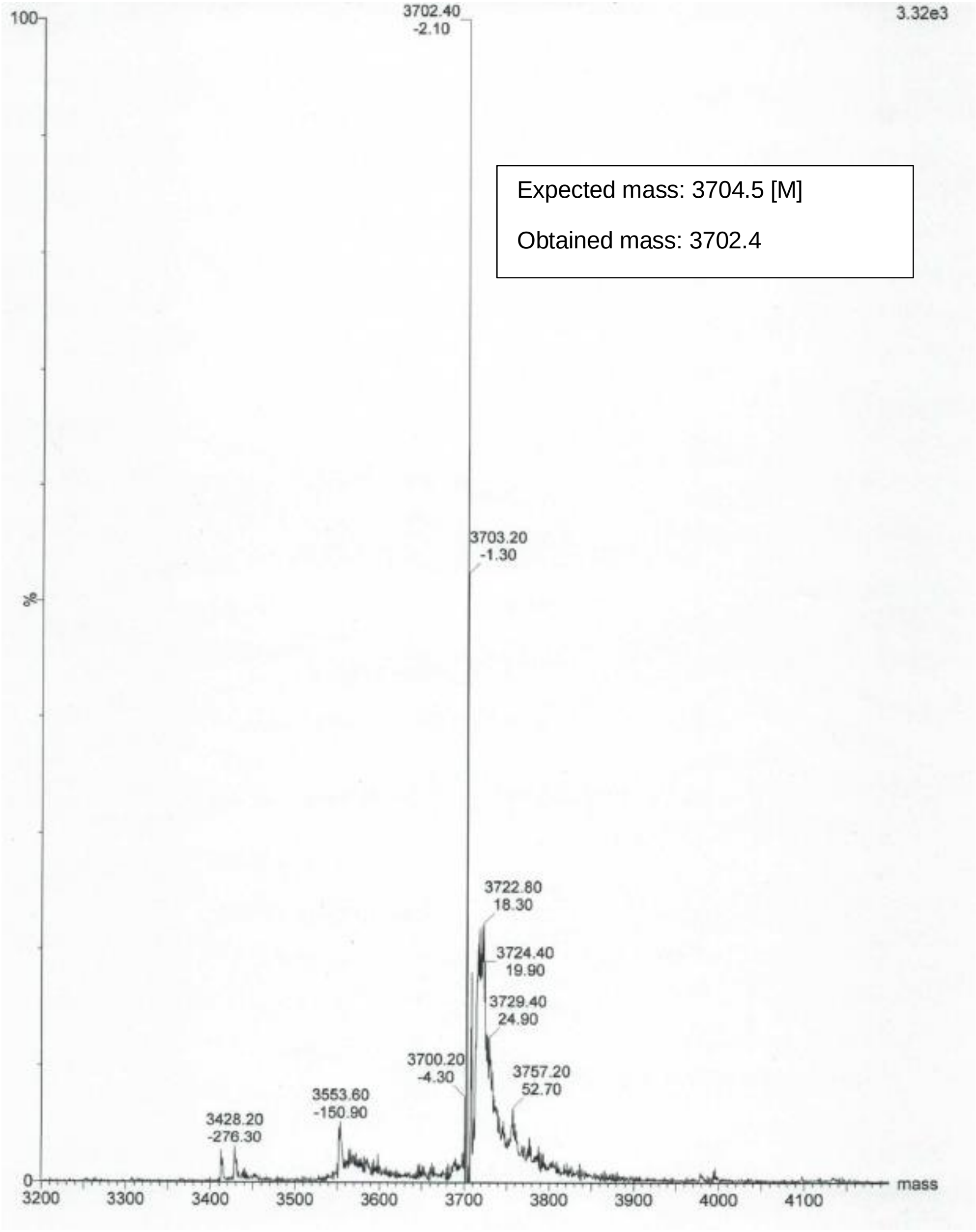

Supplementary Figure 25. ESI-ToF mass spectra for mini-PEG γ-modified oligomer P2.

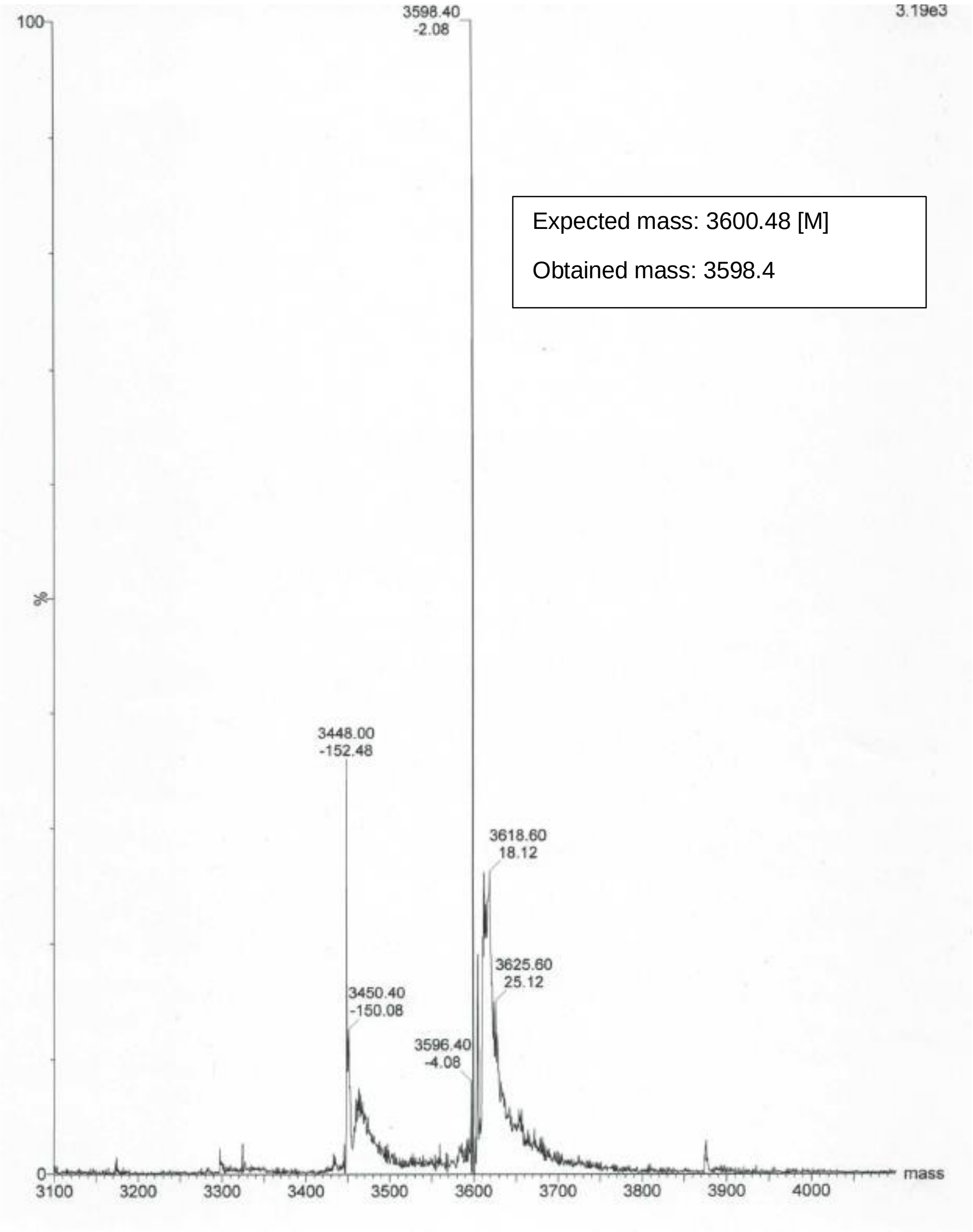

Supplementary Figure 26. ESI-ToF mass spectra for mini-PEG γ-modified oligomer P3.

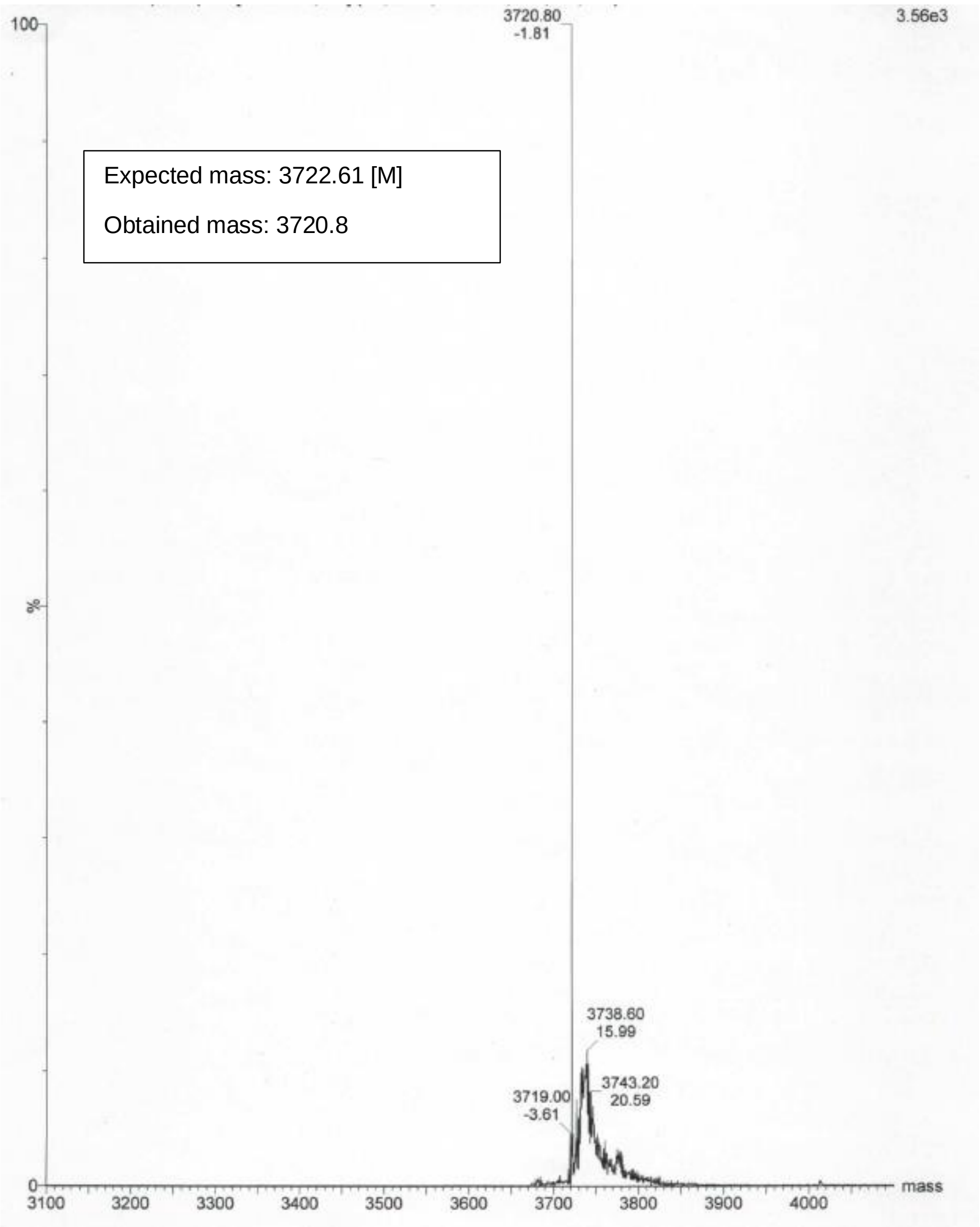

Supplementary Figure 27. ESI-ToF mass spectra for mini-PEG γ-modified oligomer P4.

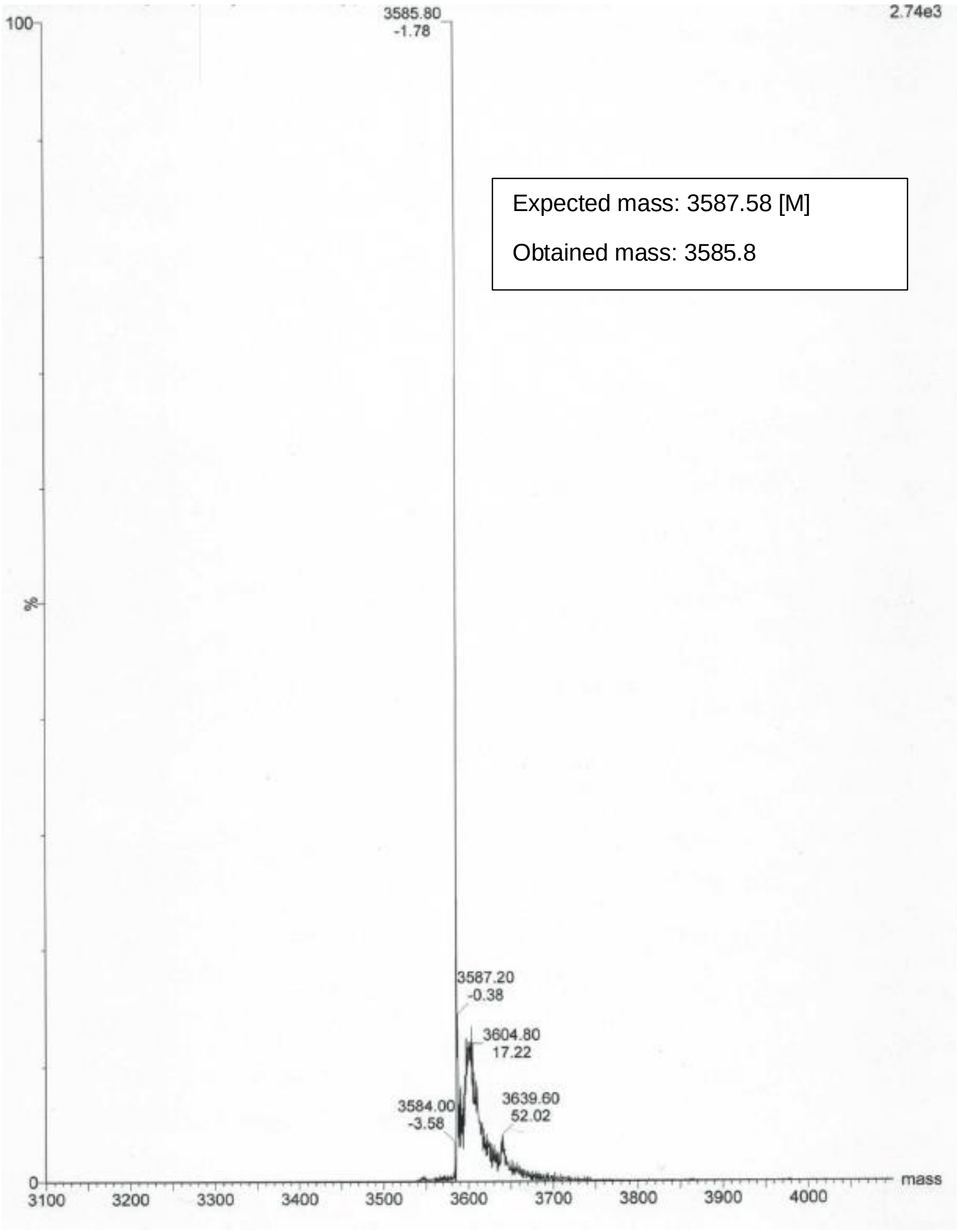

Supplementary Figure 28. ESI-ToF mass spectra for mini-PEG γ-modified oligomer P5.

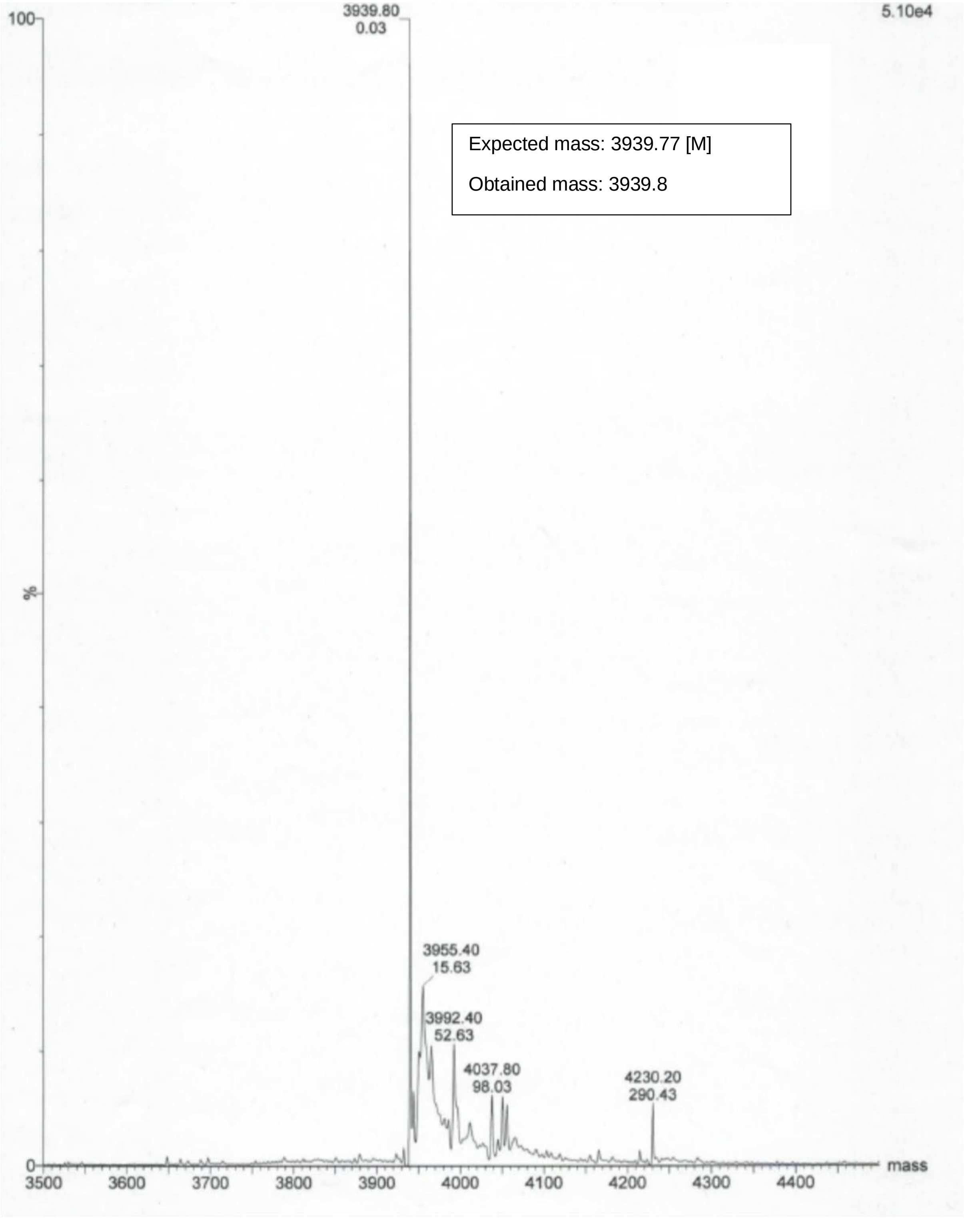

Supplementary Figure 29. ESI-ToF mass spectra for mini-PEG γ-modified oligomer P6-biotin.

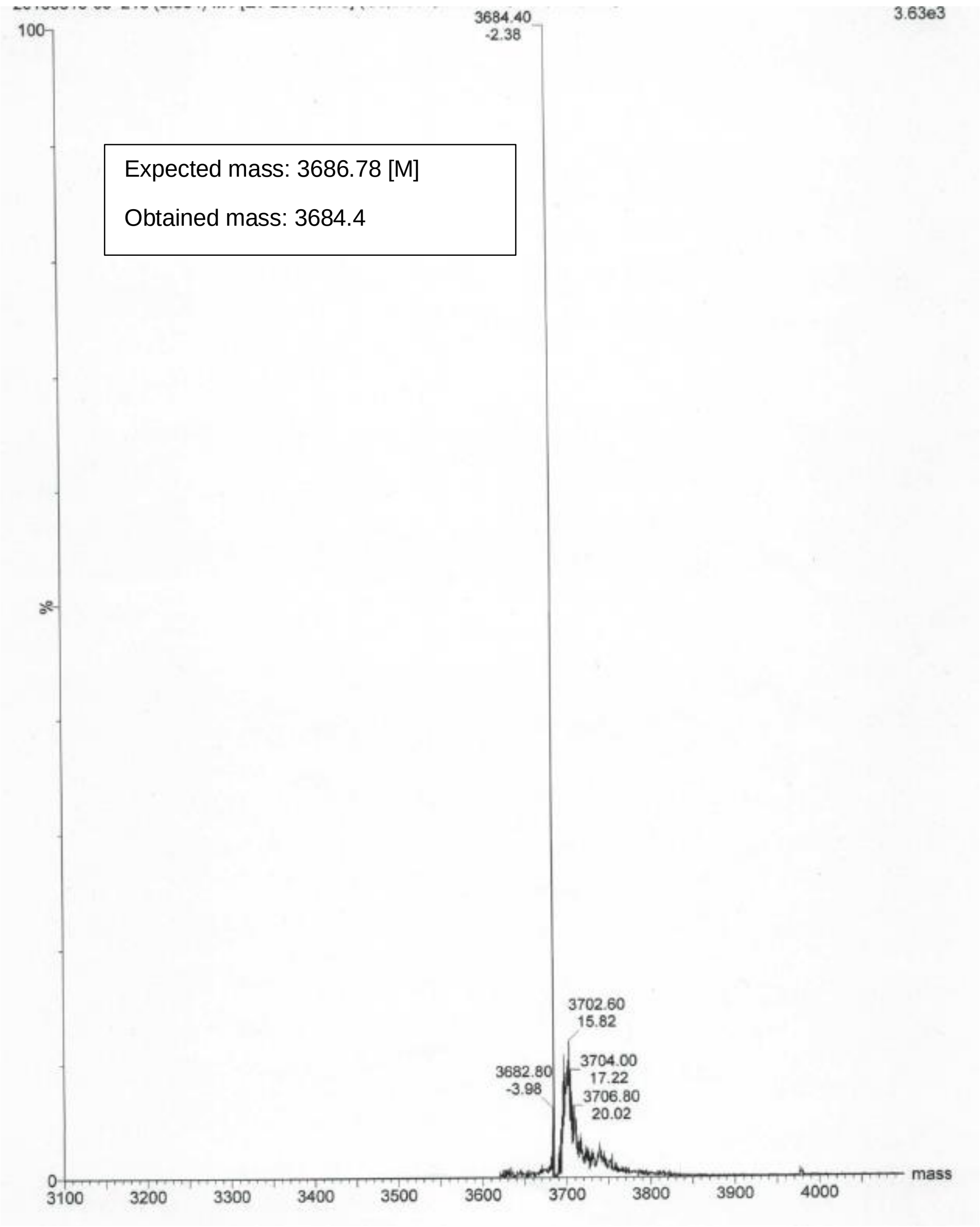

Supplementary Figure 30. ESI-ToF mass spectra for mini-PEG γ-modified oligomer P7.

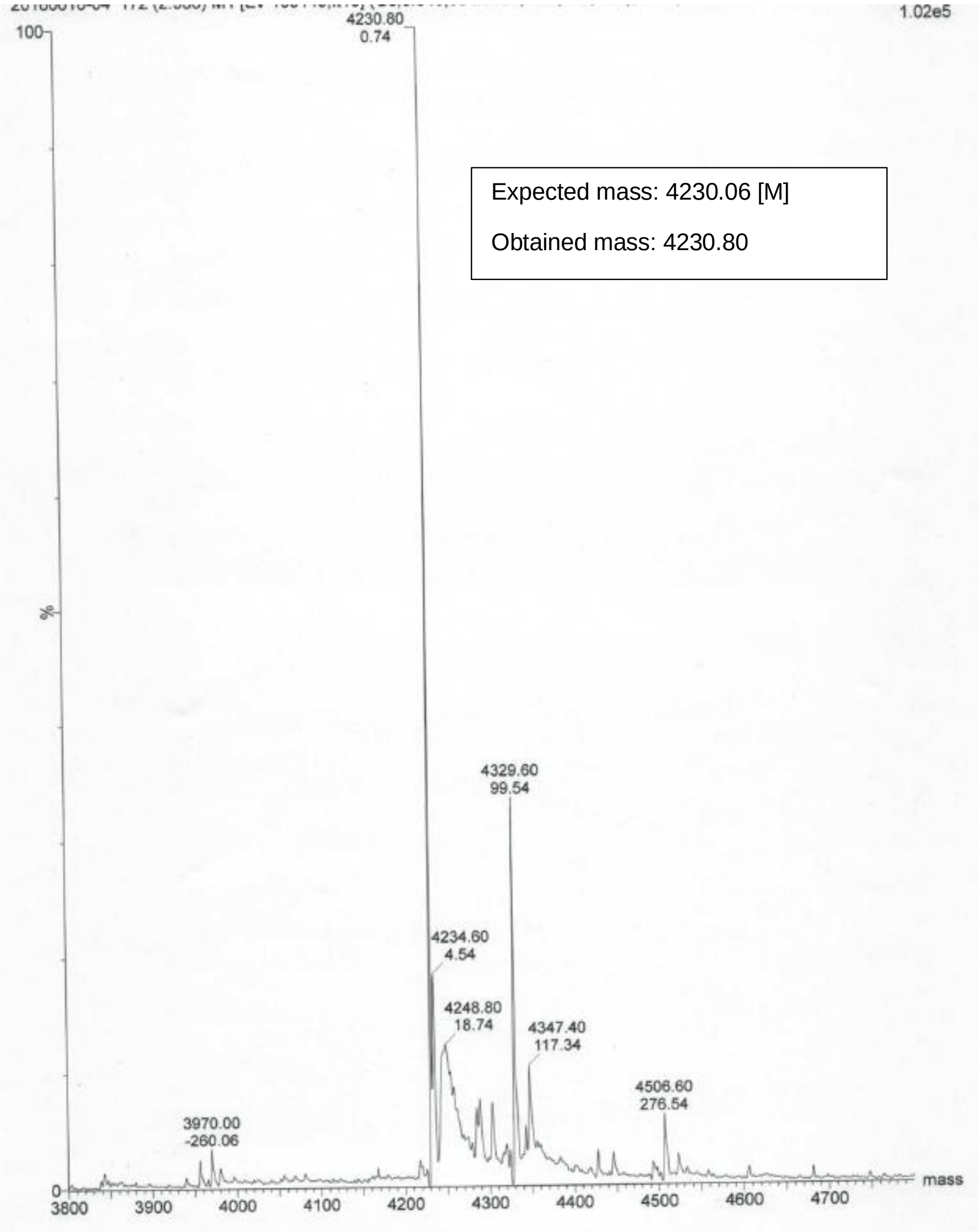

Supplementary Figure 31. ESI-ToF mass spectra for mini-PEG γ-modified oligomer P8-Cy3.

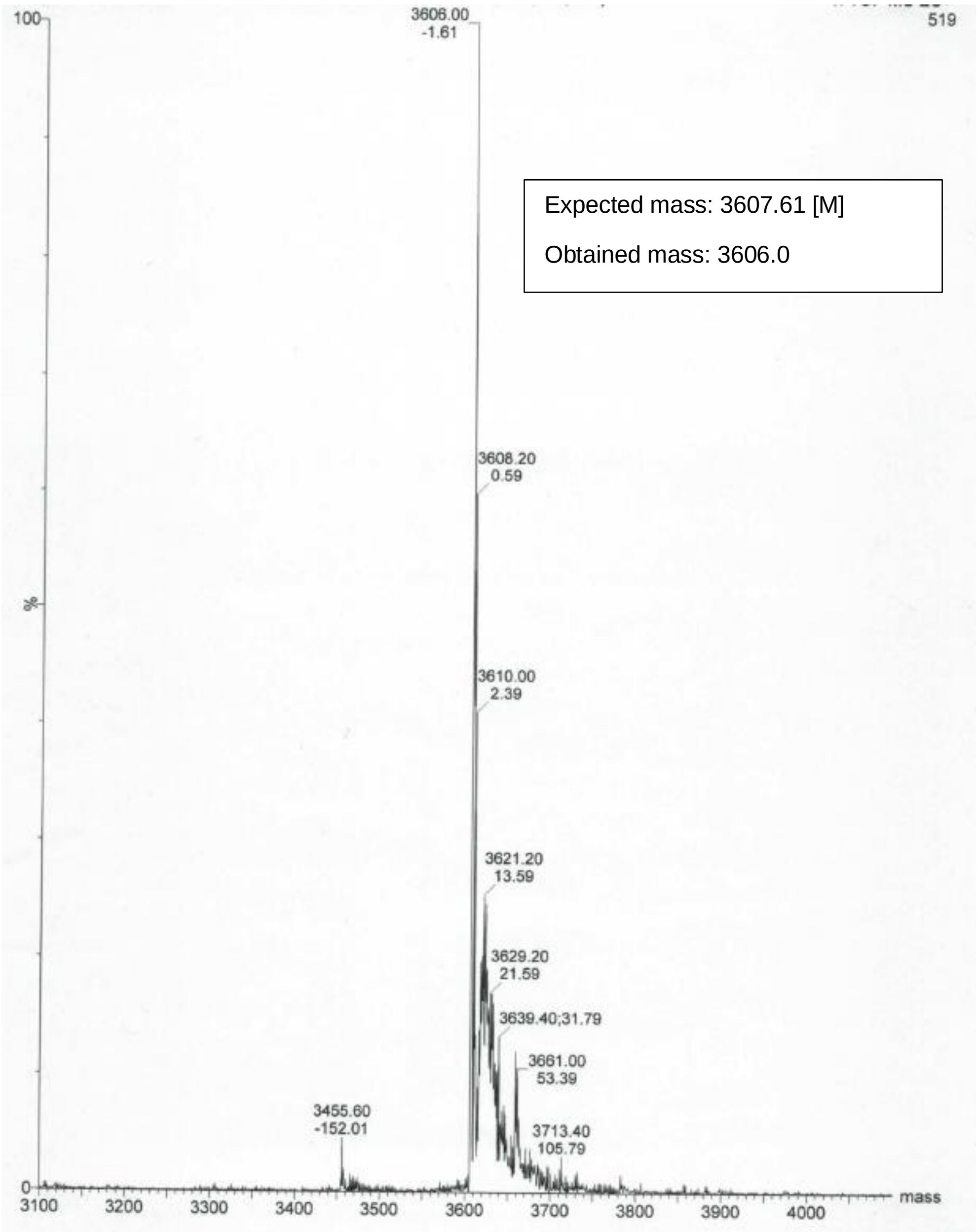

Supplementary Figure 32. ESI-ToF mass spectra for mini-PEG γ-modified oligomer P9.

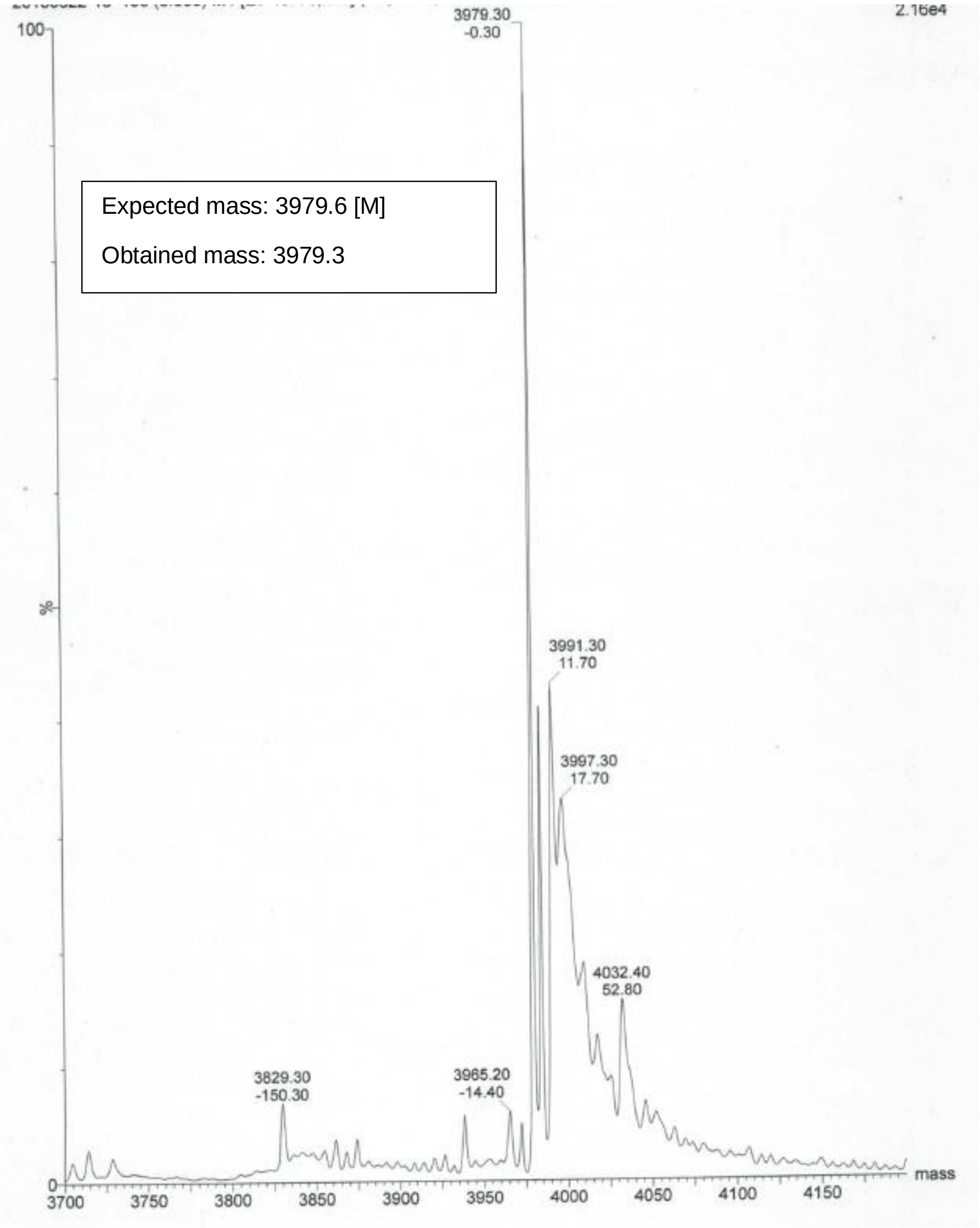

(j)

Supplementary Figure 33. ESI-ToF mass spectra for mini-PEG γ-modified oligomer P2m.

Supplementary References

1) Dirks, R.M., Lin, M., Winfree, E., Pierce N.A. Paradigms for computational nucleic acid design. *Nucleic Acids Res.* **32**(4):1392-403 (2004).

2) Hariadi, R.F *et al.* Mechanical coordination in motor ensembles revealed using engineered artificial myosin filaments. *Nat. Nanotechnol.*, **10**(8):696–700, 08 (2015)

3) Yang, Y. *et al.* Self-Assembly of DNA Rings from Scaffold-Free DNA Tiles. *Nano Lett.,* **13**, 4, 1862-1866 (2013)

4) Meijering, E.*et al.* Design and validation of a tool for neurite tracing and analysis in fluorescence microscopy images. *Cytometry Part A: the journal of the International Society for Analytical Cytology.* **58**(2):167-76 (2004)

5) Schneider, C.A., Rasband, W.S., Eliceiri, K.W. NIH Image to ImageJ: 25 years of image analysis. *Nat Methods* **9**(7):671-5 (2012).
