# Supplementary material for: Modular self-assembly of gamma-modified peptide nucleic acids in organic solvent mixtures
Source: Nat Commun. 2020 Jun 11;11:2960. doi: 10.1038/s41467-020-16759-8 (PMC7289805; doi:10.1038/s41467-020-16759-8)
Supplement: Supplementary file 3 — Reporting Summary [file 41467_2020_16759_MOESM3_ESM.pdf]

nature research

Corresponding author(s): Rebecca E. Taylor

Last updated by author(s): May 8, 2020

Reporting Summary

Nature Research wishes to improve the reproducibility of the work that we publish. This form provides structure for consistency and transparency in reporting. For further information on Nature Research policies, see our Editorial Policies and the Editorial Policy Checklist.

Statistics

For all statistical analyses, confirm that the following items are present in the figure legend, table legend, main text, or Methods section.

n/a	Confirmed	
☐	☒	The exact sample size (n) for each experimental group/condition, given as a discrete number and unit of measurement
☐	☒	A statement on whether measurements were taken from distinct samples or whether the same sample was measured repeatedly
☒	☐	The statistical test(s) used AND whether they are one- or two-sided<br>*Only common tests should be described solely by name; describe more complex techniques in the Methods section.*
☒	☐	A description of all covariates tested
☐	☒	A description of any assumptions or corrections, such as tests of normality and adjustment for multiple comparisons
☐	☒	A full description of the statistical parameters including central tendency (e.g. means) or other basic estimates (e.g. regression coefficient) AND variation (e.g. standard deviation) or associated estimates of uncertainty (e.g. confidence intervals)
☒	☐	For null hypothesis testing, the test statistic (e.g. F, t, r) with confidence intervals, effect sizes, degrees of freedom and P value noted<br>*Give P values as exact values whenever suitable.*
☒	☐	For Bayesian analysis, information on the choice of priors and Markov chain Monte Carlo settings
☒	☐	For hierarchical and complex designs, identification of the appropriate level for tests and full reporting of outcomes
☒	☐	Estimates of effect sizes (e.g. Cohen's d, Pearson's r), indicating how they were calculated

Our web collection on statistics for biologists contains articles on many of the points above.

Software and code

Policy information about availability of computer code

Data collection	Nikon NIS-Elements software (version 5.20.01) was used for fluorescence microscopy image acquisition. Varian Cary WinUV package (version 4.10) with thermal software was used for melt curve data collection. TEM image acquisition was performed using Advanced Microscopy Techniques (AMT) capture engine software (version 602.600.35)
Data analysis	FIJI (ImageJ, version 2.0) was used for image analysis and export. Microsoft Excel (16.16.2) and Minitab (19.1.1) were used for mathematical analysis and graphing of microscopy and melting curve data

For manuscripts utilizing custom algorithms or software that are central to the research but not yet described in published literature, software must be made available to editors and reviewers. We strongly encourage code deposition in a community repository (e.g. GitHub). See the Nature Research guidelines for submitting code & software for further information.

Data

Policy information about availability of data

All manuscripts must include a data availability statement. This statement should provide the following information, where applicable:

- Accession codes, unique identifiers, or web links for publicly available datasets
- A list of figures that have associated raw data
- A description of any restrictions on data availability

The source data that support the findings of Figs. 1c, 2b, 2d, 4c, and the Supplementary Figs. 1, 2, 3a, 7, 9 and Supplementary table 3 are included in the Source Data file. Additionally, data repository that include raw and processed TIRF images for different conditions can be accessed from 10.6084/m9.figshare.12269288. Data repository that include raw TEM images can be accessed from 10.6084/m9.figshare.12269297. Other additional data are available from the corresponding author upon reasonable request.

Field-specific reporting

Please select the one below that is the best fit for your research. If you are not sure, read the appropriate sections before making your selection.

☒ Life sciences ☐ Behavioural & social sciences ☐ Ecological, evolutionary & environmental sciences

For a reference copy of the document with all sections, see nature.com/documents/nr-reporting-summary-flat.pdf

Life sciences study design

All studies must disclose on these points even when the disclosure is negative.

Sample size	Sample sizes were chosen for analysis of TIRF and TEM data. Sample sizes were chosen as visibly separate nanostructures under each condition over independently separate experiments as mentioned in the figure legends. For TEM studies, samples were chosen to understand the propensity of nanofibers to bundle along their width. Choice of sample size ranges were based on minimum size ranges described in the Nature Methods article titled "Visualizing samples with box plots" (https://www.nature.com/articles/nmeth.2813.pdf?origin=ppub) which describes a minimum N=30 for histogram plots (and N = 5 for box-whisker plots). We therefore chose sample size ranges of 30-200 for width analyses (TEM) for corresponding descriptive statistics studies.<br>For TIRF studies, choice of sample size ranges were based on sample size ranges [100-500 for length analyses using fluorescence microscopy] of similar nanotube growth strategy established for DNA nanotechnology as shown in the publication titled "Programming DNA tube circumferences". (DOI: 10.1126/science.1157312)
Data exclusions	For length analyses using TIRF images, a threshold of 2µm was applied to exclude visible aggregates and optical effects caused by DMSO particular to our study for identifying visibly clear and separate nanofibers. A previous study titled "Ensemble Force Changes that Result from Human Cardiac Myosin Mutations and a Small-Molecule Effector" on fluorescent filamentous F-actin systems have applied filtering/ thresholding on length for fluorescent microscope analysis (https://doi.org/10.1016/j.celrep.2015.04.006)
Replication	Microscopy experiments were performed over the course of several months and were successfully reproduced. The number of individually repeated experiments have been mentioned in the figure legends for each figure wherever applicable as well as in the "Statistics and reproducibility" section. Images were also acquired from various locations of the microchannel for TIRF and TEM grids for TEM studies to ensure reproducibility. This is commonly established in the field of DNA nanotechnology as shown in Nature communications article titled "Sites of high local frustration in DNA origami" (https://www.nature.com/articles/s41467-019-09002-6) for AFM experiments on mica. Melting experiments were repeated twice to ensure the resulting melting temperature results differed no more than 1 degree Celsius.
Randomization	No randomization was required for the experiments performed within. This is because this study focuses on the bottom-up self-assembly of nanostructures where the studies do not differ in a systematic way to allow for any bias.
Blinding	No blinding was required for the experiments performed within as this study does not involve any behavioral or clinical research where such a study would be warranted.

Reporting for specific materials, systems and methods

We require information from authors about some types of materials, experimental systems and methods used in many studies. Here, indicate whether each material, system or method listed is relevant to your study. If you are not sure if a list item applies to your research, read the appropriate section before selecting a response.

Materials & experimental systems

n/a	Involved in the study	
☒	☐	Antibodies
☒	☐	Eukaryotic cell lines
☒	☐	Palaeontology and archaeology
☒	☐	Animals and other organisms
☒	☐	Human research participants
☒	☐	Clinical data
☒	☐	Dual use research of concern

Methods

n/a	Involved in the study	
☒	☐	ChIP-seq
☒	☐	Flow cytometry
☒	☐	MRI-based neuroimaging
